# Cytosolic proteins can exploit membrane localization to trigger functional assembly

**DOI:** 10.1371/journal.pcbi.1006031

**Published:** 2018-03-05

**Authors:** Osman N. Yogurtcu, Margaret E. Johnson

**Affiliations:** Department of Biophysics, Johns Hopkins University, Baltimore, MD, United States of America; University of Virginia, UNITED STATES

## Abstract

Cell division, endocytosis, and viral budding would not function without the localization and assembly of protein complexes on membranes. What is poorly appreciated, however, is that by localizing to membranes, proteins search in a reduced space that effectively drives up concentration. Here we derive an accurate and practical analytical theory to quantify the significance of this dimensionality reduction in regulating protein assembly on membranes. We define a simple metric, an effective equilibrium constant, that allows for quantitative comparison of protein-protein interactions with and without membrane present. To test the importance of membrane localization for driving protein assembly, we collected the protein-protein and protein-lipid affinities, protein and lipid concentrations, and volume-to-surface-area ratios for 46 interactions between 37 membrane-targeting proteins in human and yeast cells. We find that many of the protein-protein interactions between pairs of proteins involved in clathrin-mediated endocytosis in human and yeast cells can experience enormous increases in effective protein-protein affinity (10–1000 fold) due to membrane localization. Localization of binding partners thus triggers robust protein complexation, suggesting that it can play an important role in controlling the timing of endocytic protein coat formation. Our analysis shows that several other proteins involved in membrane remodeling at various organelles have similar potential to exploit localization. The theory highlights the master role of phosphoinositide lipid concentration, the volume-to-surface-area ratio, and the ratio of 3D to 2D equilibrium constants in triggering (or preventing) constitutive assembly on membranes. Our simple model provides a novel quantitative framework for interpreting or designing *in vitro* experiments of protein complexation influenced by membrane binding.

## Author summary

In a multitude of cellular processes, including cell division and endocytosis, proteins must bind to one another to form large multi-protein complexes. To initiate the formation of these critical multi-protein assemblies at the right time and the right place, the constituent proteins must be present at sufficient concentrations. We show here that membrane localization offers a powerful way of controlling protein concentrations by reducing the dimensionality of the protein’s search space. We present a simple and practical analytical theory that determines the significance of membrane localization for triggering protein-protein interactions. We show that protein binding partners will often form substantially more complexes when both partners can localize to surfaces, and thus localization can regulate the timing of multi-protein assembly. We collect *in vitro* binding data and cellular concentrations of proteins and lipids involved in pathways including clathrin-mediated endocytosis to demonstrate how cellular proteins could exploit membrane localization to regulate assembly.

## Introduction

When clathrin, the essential cytosolic protein of clathrin-mediated endocytosis (CME), self-assembles into multi-protein cages, the same protein-protein contacts are used regardless of whether clathrin is in solution or on the membrane [1–3]. However, more binding [2] is observed on the membrane. A fundamental phenomenon for explaining this change is dimensionality reduction: if proteins on the membrane search a smaller space, then this increases their relative concentration; higher concentration of proteins (reactants) shifts the equilibrium to produce more protein-protein complexes (products) as defined by LeChatelier’s principle. The question we address is, how significant a role can this dimensionality reduction play for driving protein-protein interactions between cytosolic proteins *in vitro*? Understanding this role can help interpret mechanisms of assembly *in vivo*. Despite the wide-ranging cellular processes such as cell division and viral budding that could exploit this phenomenon, it has so far lacked a predictive theoretical framework. Hence while the concept that membrane localization can enhance binding may be familiar or intuitive, we here make that concept quantitative for soluble binding partners. In contrast, theory for understanding reduction of dimensionality in chemoreception and receptor mediated signaling (where it can also be functionally significant [4]) has been studied for decades [5, 6]. Membrane localization can accelerate a ligand’s search for membrane bound targets [5–9] and increase activation of intracellular receptors, influencing downstream response [8–10]. However, in these cases, a soluble protein always targets a membrane bound receptor. Here we capture the dynamic cases where both binding partners are soluble and target lipids present in limited concentrations, as occurs, for example, in CME. Our theory determines how binding enhancement depends on protein and lipid concentrations, protein-protein and protein-lipid affinities, the volume-to-surface area ratio, and the change in binding affinities from 3D to 2D. Quantifying this behavior is critical to understanding assembly on surfaces because 2D localization can strengthen binding reactions regardless of whether additional factors, such as curvature generation [11], membrane microdomains [12, 13], or conformational switches [1], also influence binding.

**Fig 1:**
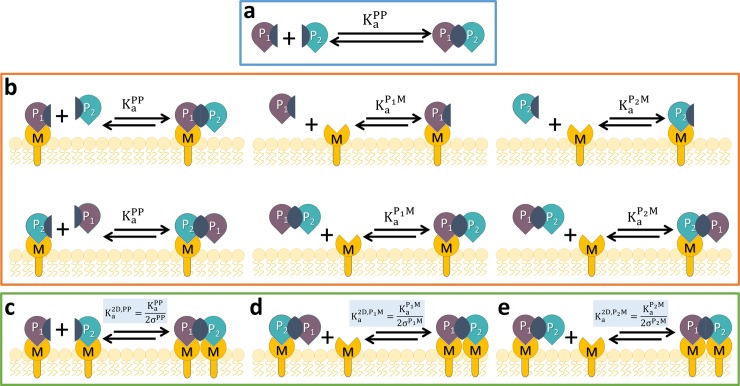
Quantifying how protein binding partners in solution can increase complex formation by binding to lipids on membrane surfaces. This model contains two types of proteins (P_1_ and P_2_) and one type of lipid (M). We show all ten possible binding interactions that can occur between this pair of proteins that bind each other (P_1_+P_2_⇌P_1_P_2_) and also bind specific lipids (M), producing a system of nine total distinct species: P_1_, P_2_, M, P_1_P_2_, MP_1_, P_2_M, MP_1_P_2_, P_1_P_2_M, and MP_1_P_2_M. a) Solution (3D) binding. b) Interactions in solution (3D) that pull proteins to the membrane surface through protein or lipid binding. In (c-e) the binding interactions are in 2D (species concentration in Area^-1^) and can exploit the lower search space. Conversion from 3D to 2D equilibrium constant is defined by the variable σ, where only σ = σ^PP^ appears in Eq 3. To solve for all species in consistent units (i.e. Volume^-1^), K_a_^2D^ values must be multiplied by V/A. The size of solution volume V vs. membrane surface area A is thus a critical parameter controlling binding enhancement. The membrane surface can be the plasma membrane, for example, but also liposomes suspended in solution. d,e) Proteins localized at the surface will also bind lipids in a 2D search. There are over 100 functionally diverse peripheral membrane proteins in yeast alone [14] whose binding interactions with one another could strengthen substantially via binding to membranes. We simulated this model for a comprehensive range of conditions using mainly systems of ordinary differential equations (ODE), but also single-particle reaction-diffusion (RD) simulations [19, 20] (Methods).

We show here that membrane localization offers a powerful way of controlling protein concentrations and therefore of regulating the timing of multi-protein assembly. In many cases, we find that the power of membrane localization to drive binding is highly robust; strong and weak protein-protein interactors, at high or low concentrations, will all benefit significantly from membrane localization. The analytical theory we present describes a relatively simple model at equilibrium where a pair of soluble binding partners can form complexes in solution and also can both bind and continue to form complexes on the surface of a membrane (Fig 1). Thus it is useful as a tool to quantify protein-protein interactions that, while physiologically relevant, are being studied *in vitro*. Without accounting for the complex array of factors present *in vivo*, such as variability in membrane composition, competition for protein and lipid binding from diverse proteins, spatial distributions of proteins or lipids, and non-equilibrium dynamics, we can only speculate about the behavior in the cell. However, the theory provides a novel and valuable metric for interpreting how important membrane localization can be given the concentrations and binding properties of component proteins, and it isolates the role of membrane localization from other factors. Since even most *in vitro* experiments contain more components and complexity than is captured in our simple model, we discuss how it can still be used as a quantitative guide for estimating how membrane heterogeneity, competition for binding, and mutations would influence the parameters of the model (volume, surface area, binding affinities and concentrations) and thus the proteins’ subsequent response to localization. We specifically address in our results how lipids such as PI(4,5)P_2_ can be targeted by many proteins at any time [14, 15], how some membrane binding domains such as BAR domains bind membranes with widely varying lipid composition and in a curvature dependent manner [16, 17], and how mutations and multiple protein binding partners would alter protein complex formation. Despite the limitations of applying an equilibrium theory to understand complexation that ultimately occurs in the nonequilibrium cell, we believe the theory represents a well-defined starting point from which to probe more complex systems, just as using *in vitro* studies provide a useful guide for interpreting behavior in the cell. It is also a reference point for studying the time-dependence of assembly through computer simulation, as we do here, and a starting point from which to build further complexity into the model.

We apply the theory here to characterizing, within a quantitative framework, the role of membrane localization for enhancing 55 binding interactions involving 33 distinct protein pair interaction sets (S1 Table). Through simulation, we also move beyond the model illustrated in Fig 1 of only pairs of soluble binding partners to show how complexation involving non-membrane binding scaffold proteins such as clathrin, or how formation of higher-order oligomers, which is functionally important for driving membrane remodeling [11, 17, 18], can also be regulated by membrane localization (13 additional interaction sets in S2 Table). Our theory only applies to the pair interactions illustrated in Fig 1. We include 22 proteins involved in CME in both human and yeast cells, as well as 15 proteins involved in lipid regulation, vesicle formation on endosomes, budding, and morphogenesis in yeast cells (Table 1). We collected concentration and cellular geometry data based on *in vivo* values to better connect to physiologic regimes (S3 Table and S4 Table). Although our theory represents an approximate solution to the full model shown in Fig 1, we show through extensive simulations using both systems of ordinary differential equations and single-particle reaction-diffusion, [19, 20] that it is highly accurate. Through simulation, we additionally find that membrane localization alters the timescales of protein-protein assembly, but that the result is not dominated by changes in protein diffusion between solution and the membrane. Rather, for physiologic binding strengths, the rate-limiting step is the speed of binding to the membrane surface from solution. Finally, a practical application of our simple formula is that it can be used to experimentally fit protein-protein binding affinities on surfaces (K_a_^2D^), which are rarely measured [21, 22]. The advantage of the formula is that it applies to *in vitro* experiments where a pair of proteins can *reversibly* bind to the membrane, thus avoiding the need to restrict proteins to the surface.

**Table 1:** Proteins studied, copy numbers, protein-lipid interactions (PLI) and affinities (K_d_^PM^).

	Protein	Species	Copy Number^d^	K_d_^PM^ (μM)	Literature Refs
1	OSH2	Yeast	850	1–1.5	PLI: PMID:11238399. Affinity, measured: same ref.
2	SWH1	Yeast	505	3.5–6.2	PLI: PMID:21119626. Affinity, measured: same ref.
3	KES1	Yeast	21166	0.055–2.2	PLI: PMID:22162133,11916983. Affinity, measured: same.
4	VPS17	Yeast	1077	>100	PLI: PMID:11557775. Affinity, measured: same ref.
5	SNX4	Yeast	1483	>100	PLI: PMID:11557775. Affinity, measured: same ref.
6	SNX41	Yeast	367	>100	PLI: PMID:11557775. Affinity, measured: same ref.
7	VPS5	Yeast	1326	>100	PLI: PMID:11557775. Affinity, measured: same ref.
8	ATG20	Yeast	519	>100	PLI: PMID:11557775. Affinity, measured: same ref.
9	BOI2	Yeast	567	6.6–19.5	PLI: PMID:15023338. Affinity, measured: same ref.
10	CLA4	Yeast	397	20.2–100	PLI: PMID:15023338. Affinity, measured: same ref.
11	SKM1	Yeast	16	3.9–6.4	PLI: PMID:15023338. Affinity, measured: same ref.
12	BEM1	Yeast	1037	>100	PLI: PMID:11557775. Affinity, measured: same ref.
13	BOI1	Yeast	399	20	PLI: PMID:15023338. Affinity, measured: same ref.
14	VAM7	Yeast	210	2–3	PLI: PMID:11557775. Affinity, measured: same ref.
15	SNX3	Yeast	5092	2–3	PLI: PMID:11557775. Affinity, measured: same ref.
16	SLA2	Yeast	3904	0.27–3.4	PLI: PMID:15574875. Affinity, homology (AP180): PMID: 12740367.
17	SYP1	Yeast	2467	Used 0.1, 10, 100	PLI: PMID:19713939,1321812. Affinity, not known, estimated.
18	ENT1	Yeast	1750	0.08	PLI: PMID:22193158,10449404. Affinity, homology (EPN1): PMID:17825837.
19	ENT2	Yeast	1325	0.08	PLI: PMID:22193158,10449404. Affinity, homology (EPN1): PMID:17825837.
20	YAP1802	Yeast	264	0.27–3.4	PLI: PMID:22193158,21119626. Affinity, homology (AP180): PMID:12740367.
	CHC1 (Not Studied)	Yeast	19278^a^	No binding	-
21	SLA1	Yeast	2964	No binding	-
22	EDE1	Yeast	5964	No binding	-
23	FCHO1	Human	3706	Used 0.1, 10, 100	PLI: PMID:22484487. Affinity, not known, estimated.
24	AP-2	Human	244537^b^	2.86 (0.072)	PLI: PMID:15916959. Affinity, measured: same ref.
25	EPN1	Human	570949	0.08	PLI: PMID:17825837. Affinity, measured: same ref.
26	PICALM	Human	358673	2.7–3.4	PLI: PMID:25090048. Affinity, homology (AP180): PMID:12740367.
27	DAB2	Human	1078162	0.08	PLI: PMID:12234931. Affinity, homology (EPN1): PMID:17825837.
28	FCHO2	Human	36302	Used 0.1, 10, 100	PLI: PMID:20448150. Affinity, not known, estimated.
29	SNAP91/AP180	Human	21716^c^	0.27–3.4	PLI: PMID:12740367. Affinity, measured: same ref.
30	LDLRAP1/ARH	Human	1048	0.08	PLI: PMID:12234931. Affinity, homology (EPN1): PMID:17825837.
31	HIP1	Human	13771	0.27–3.4	PLI: PMID:14732715. Affinity, homology (AP180): PMID:12740367.
32	HIP1R	Human	24161	0.27–3.4	PLI: PMID:14732715. Affinity, homology (AP180): PMID:12740367.
33	AMPH	Human	89536	0.1	PLI: PMID:22888025. Affinity, measured: same ref.
34	SH3GL2/Endophilin	Human	55621	0.03	PLI: PMID:22888025. Affinity, measured: same ref.
35	EPS15	Human	91354	No binding	-
36	ITSN1	Human	20184	No binding	-
37	CLTC	Human	1495814^a^	No binding	-

a) To simulate clathrin trimers, the heavy chain copy numbers reported here are divided by 3.

b) Considering AP2A1 gene.

c) Based on ppm value from PMID: 24920484. We scaled this value by the number of AP-2s from PMID: 26496610 to obtain the predicted number of AP180s in the cell.

d) Copy number for yeast from Ref. [49] and humans Ref: [50]

## Results

### Model and theory

In our primary model, we consider two proteins P_1_ and P_2_, that bind in solution with equilibrium constant $K_{a}^{PP,3D}=\frac{{\left[P_{1}P_{2}\right]}_{eq}}{{\left[P_{1}\right]}_{eq}{\left[P_{2}\right]}_{eq}}=\frac{{\left[P_{1}P_{2}\right]}_{eq}}{\left({\left[P_{1}\right]}_{0}-{\left[P_{1}P_{2}\right]}_{eq})({\left[P_{2}\right]}_{0}-{\left[P_{1}P_{2}\right]}_{eq}\right)}$, where total concentrations of the proteins are [*P*_1_]_0_ = [*P*_1_*P*_2_] + [*P*_1_] and same for [P_2_]_0_. If these proteins can also reversibly bind to membranes via targeting a specific lipid *M*, and continue to bind one another, their binding equilibrium will shift as a total of nine distinct species can form (Fig 1, Methods). The bound protein-protein complexes can either be in solution or on the membrane, [*P*_1_*P*_2_]^*sol*+*mem*^ = [*P*_1_*P*_2_]^*sol*^ + [*P*_1_*P*_2_]^*mem*^ = [*P*_1_*P*_2_]+[*P*_1_*P*_2_*M*]+[*MP*_1_*P*_2_] [*MP*_1_*P*_2_*M*], and unbound species are similarly defined [*P*_1_]^*sol*^ + [*P*_1_]^*mem*^ = [P_1_]+[MP_1_], and [*P*_2_]^*sol*^ + [*P*_2_]^*mem*^ = [P_2_]+[P_2_M], where *M* indicates a copy of a target membrane lipid bound to P_1_ or P_2_. The model thus assumes each protein binds membrane via targeting a single copy of a specific lipid type. Proteins on the membrane must be able to diffuse to bind one another, which is consistent with experimental observations [23] even of RNA-protein complexes (>6000kDa) that are anchored via multiple lipid binding sites along with myristoyl groups [24]. Each of the nine distinct species will be constrained to preserve detailed balance at equilibrium, as defined by the 10 pairwise binding interactions of Fig 1 (see Methods), and the total concentrations of proteins is fixed at the same values as above, but now [*P*_1_]_0_ = [*P*_1_*P*_2_]^*sol*+*mem*^+[*P*_1_]^*sol*+*mem*^, and the same for [*P*_2_]_0_. Similarly, [*M*]_0_ = [*M*]+[*MP*_1_]+[*P*_2_*M*]+[*P*_1_*P*_2_*M*]+[*MP*_1_*P*_2_]+2[*MP*_1_*P*_2_*M*]. We note that species on the membrane have concentrations normally of μm^-2^, matching the units of equilibrium constants in 2D (K_a_^2D^)^-1^. All species copy numbers, whether on or off the membrane, however, can be solved for in volume units when the appropriate Solution volume/Membrane surface Area (V/A) conversion factor scales the 2D binding constants, so we always report volume units for concentrations. To quantify the change in bound protein-protein complexes as a function of membrane localization we will define an effective equilibrium constant

$$K_{a}^{eff}=\frac{\left({\left[P_{1}P_{2}\right]}_{eq}^{sol}+{\left[P_{1}P_{2}\right]}_{eq}^{mem}\right)}{({\left[P_{1}\right]}_{eq}^{sol}+{\left[P_{1}\right]}_{eq}^{mem})\left({\left[P_{2}\right]}_{eq}^{sol}+{\left[P_{2}\right]}_{eq}^{mem}\right)}=\frac{\left({\left[P_{1}P_{2}\right]}_{eq}^{sol+mem}\right)}{\left({\left[P_{1}\right]}_{0}-{\left[P_{1}P_{2}\right]}_{eq}^{sol+mem}\right)\left({\left[P_{2}\right]}_{0}-{\left[P_{1}P_{2}\right]}_{eq}^{sol+mem}\right)}.$$ (1)

This is not a true equilibrium constant, as both bound and unbound states as defined above contain several species that do not all stepwise interconvert with one another. However, from K_a_^eff^ and initial protein concentrations [*P*_1_]_0_ and [*P*_2_]_0_, one can immediately solve for bound complex concentration using Eq 1. If proteins cannot bind to the membrane, the value of K_a_^eff^ will revert to the solution bound value, K_a_^PP^, and thus the ratio of K_a_^eff^ / K_a_^PP^ determines the extent to which membrane localization either enhances or diminishes protein-protein complex formation. As we discuss further below, in the extreme limits where all proteins are either in solution or on the membrane, K_a_^eff^ reduces to a true equilibrium constant. The strength of K_a_^eff^ is that it also quantitatively describes all the conditions in between these limits. Thus, our K_a_^eff^ definition offers a valuable metric for quantifying the equilibrium of the model in Fig 1, which must otherwise be defined by multiple quantities.

We derive below an exact expression for K_a_^eff^ based on the 10 individual equilibrium relations for each reversible binding reaction (Fig 1, Methods). The value of K_a_^eff^ for any protein pair will depend on volume V, surface area A, total protein [P_1_]_0_, [P_2_]_0_, and lipid concentrations [M]_0_, and all six true equilibrium constants between protein and lipid interactions in 3D $\left(K_{a}^{PP},K_{a}^{P_{1}M},K_{a}^{P_{2}M}\right)$ and in 2D $\left(K_{a}^{2D,PP},K_{a}^{2D,P_{1}M},K_{a}^{2D,P_{2}M}\right)$. Importantly, the K_a_^2D^ values (with units μm^2^/mol, e.g.) are different from the corresponding 3D values, but they are related through K_a_^2D^ = K_a_^3D^/(2σ). The variable σ, with units of length, is a thermodynamic property of each binding pair that captures changes to binding free energy as a result of surface restriction and changes in standard state units. Changes to free energy are largely entropic, due to altered rotational freedom and protein flexibility [22], although limitations on the orientation of the binding interfaces could alter the enthalpy. The bending rigidity of the membrane can also affect σ, by controlling the relative orientations that the binding pairs can adopt [25]. The variable σ thus represents an independent variable that is specific to each protein pair studied. It is possible that even if concentrations increase on the membrane surface, a decrease in K_a_^2D^ will cause less complex formation, and we quantify this regime in the Results section. To keep track of these distinct 3D and 2D equilibrium constants, we explicitly retain the 2D superscript for 2D binding, otherwise K_a_ (including K_a_^eff^) describes a 3D constant. To derive a simple analytical expression for K_a_^eff^, we input the pairwise equilibria (Methods) into Eq 1 and after canceling terms, we use the equilibrium expression

$$K_{a}^{P_{n}M}=\frac{{\left[P_{n}M\right]}_{eq}}{{\left[M\right]}_{eq}{\left[P_{n}\right]}_{eq}},$$ (2)

with *n* = 1 or 2, to complete the derivation (Methods).

**Fig 2:**
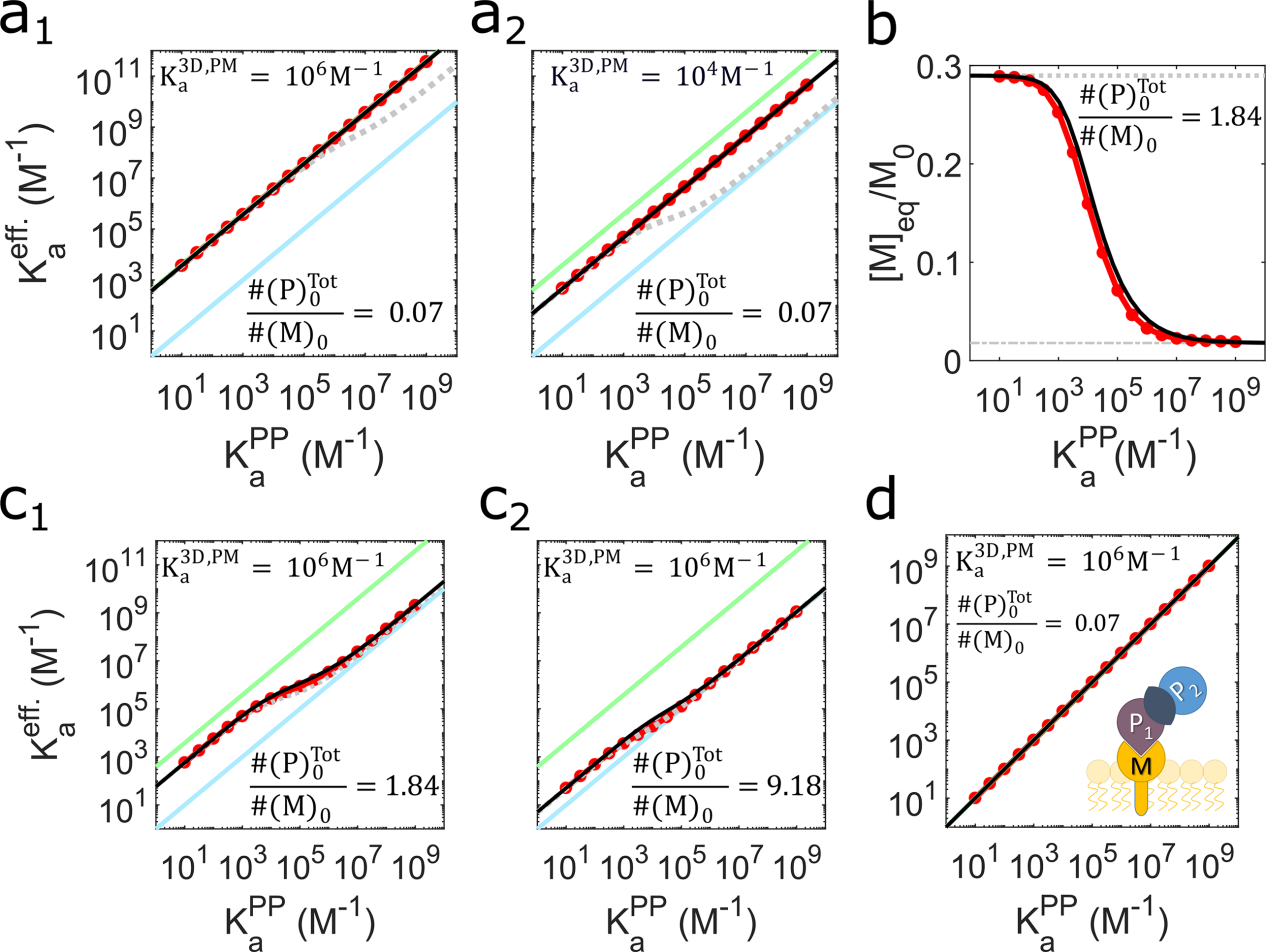
The equilibrium theory developed here accurately predicts how and when membrane localization will enhance protein interaction strength. (a) Our theory (Eq 3) shown in solid black lines in all panels, is compared with ODE simulation results shown in red circles. For reference, the blue line shows the trend for pure solution binding, i.e. K_a_^eff^ = K_a_^PP^_._ The green line shows the maximum achievable enhancement, occurring if all proteins were on the membrane, K_a_^eff^ = γK_a_^PP^. The gray dashed lines are included to contrast the K_a_^eff^ calculated using a simple approximation that lacks cooperativity (S1 Text section 2D). From a_1_ to a_2_, the K_a_^PM^ is decreased, producing lower but still constant enhancements, as the lipids are in excess relative to the proteins. (b) The number of unbound lipids is plotted as a function of K_a_^PP^ with all other parameters fixed (S1 Text section 3A), showing how lipid binding is a function of the protein interaction strength due to the cooperative effect (Fig 1D and 1E). The theoretical prediction for [M]_eq_ is shown in solid black. (c) With fewer lipid recruiters relative to total cytosolic proteins ([P]_tot_/[M]_tot_ >1), the enhancement is less pronounced, although for weak binders (low K_a_^PP^) even limited membrane localization causes significant increases in complex formation. From c_1_ to c_2_ the protein concentrations are increased. All results use σ = 1nm, see S1 Text section 3A for all other parameters. (d) If only one partner binds the membrane, the protein interaction remains fully 3D and no enhancement occurs.

Our main result is then a surprisingly simple and exact analytical relationship that quantifies the equilibrium solution of our model (Fig 1) via K_a_^eff^ and the enhancement relative to K_a_^PP^.

$$\frac{K_{a}^{eff}}{K_{a}^{PP}}=\frac{\gamma K_{a}^{P_{1}M}K_{a}^{P_{2}M}{\left({\left[M\right]}_{eq}\right)}^{2}+\left(K_{a}^{P_{1}M}+K_{a}^{P_{2}M}\right){\left[M\right]}_{eq}+1}{\left(1+K_{a}^{P_{1}M}{\left[M\right]}_{eq}\right)\left(1+K_{a}^{P_{2}M}{\left[M\right]}_{eq}\right)},$$ (3)

where γ is a dimensionless constant V/(2Aσ), σ = K_a_^PP^/2K_a_^2D,PP^, [M]_eq_ is the unbound lipid concentration at equilibrium, and all equilibrium constants (including K_a_^eff^) and concentrations are in volume units (Fig 2). [M]_eq_ is a function of all the model parameters, and can only be solved exactly using numerical methods (Methods); we therefore derive an additional approximate analytical equation for [M]_eq_ described below (Fig 2B). However, in the regime where lipids are in excess, the result of Eq 3 is particularly simple because [M]_eq_~[M]_0_, the total concentration of lipids (Fig 2A). Critically, this means that the initial experimental conditions then directly determine the enhancement. In this regime only two factors control enhancement, the ratio V/(2Aσ), and the dimensionless strengths of membrane localization, $K_{a}^{P_{n}M}{\left[M\right]}_{0}$, which report the ratio of membrane bound versus solution proteins (Eq 2) and which we term the membrane stickiness. Hence the volume-to-surface-area ratio, K_a_^PP^/K_a_^2D,PP^, and membrane stickiness play a primary role in triggering (or preventing) constitutive assembly on membranes. The right-hand side of Eq 3 is also constant for all K_a_^PP^ values (Fig 2A). In this regime, our Eq (3) can also be applied to extract binding affinities on membranes (K_a_^2D^) from experiments where binding occurs both on membranes and in solution. This practical application of our result should help simplify the relatively rare experimental characterization of protein-protein affinities on surfaces, as the proteins need not be restricted to the surface for it to work. The condition of excess lipids can be satisfied even with a lipid recruiter such as PI(4,5)P_2_, present at 2.5x10^4^μm^-2^ in the plasma membrane [15], or ~1% of lipids [13] (Fig 2A), as we explore further below for proteins involved in CME.

**Fig 3:**
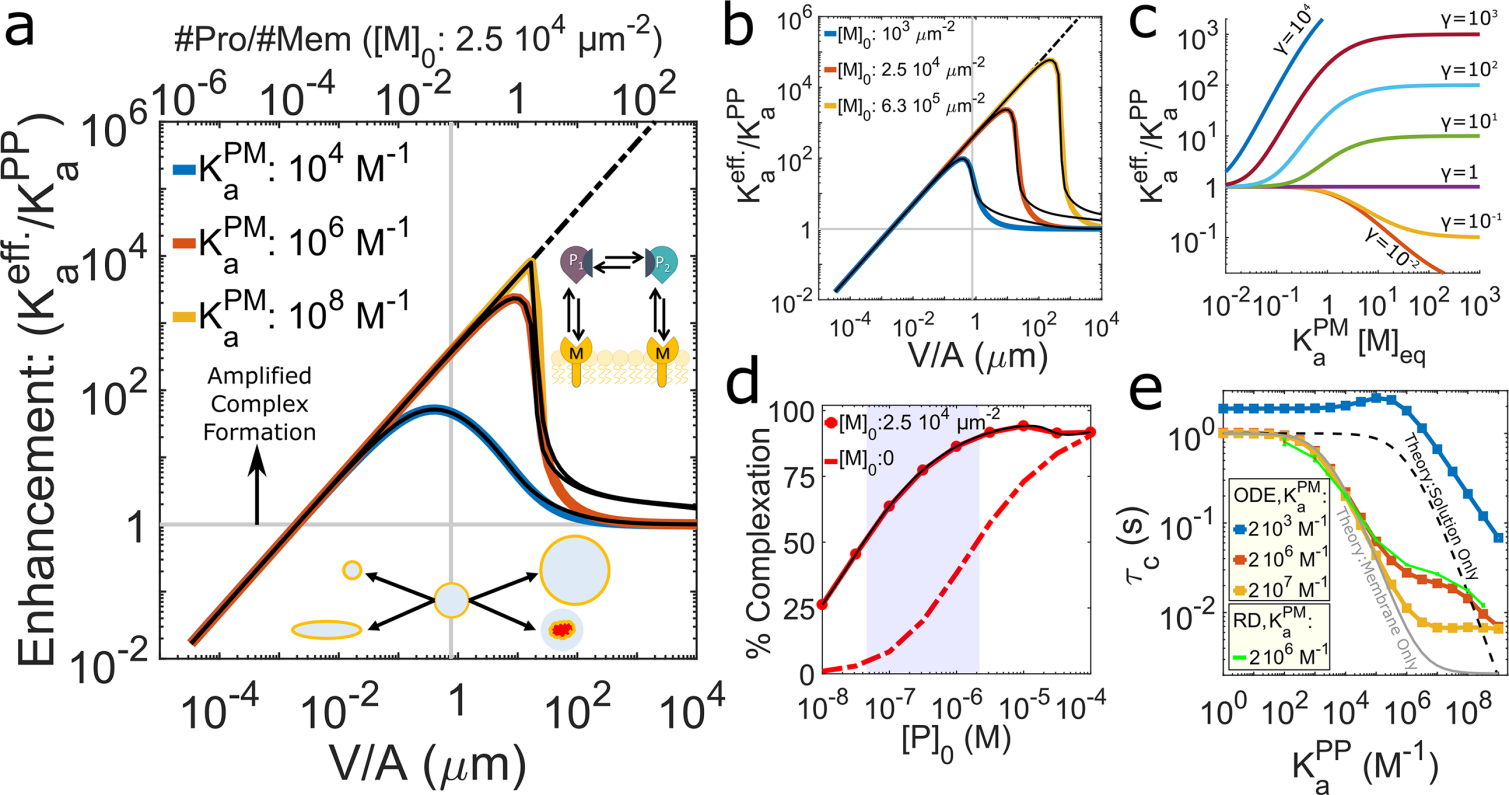
Protein interactions aided by strong protein-lipid interactions, abundant lipid recruiters, and low protein concentrations benefit most widely from membrane localization. a,b) Enhancement ratios from ODE simulation (colored lines) and theory (black solid lines in all panels). The dashed black line is the upper limit for the enhancement ratio given by K_a_^eff^/K_a_^PP^ = γ. σ = 1nm. Between the limiting cases where the membrane surface area is either too large to enhance binding (V/A⟶0) or is too small to effect binding (V/A⟶∞), a broad region of enhanced binding occurs. The vertical gray line is the V/A ratio for the yeast plasma membrane (0.5μm), for reference, and most physiologic V/A values fall in the range ~0.05–20μm (S3 Table). Within this physiologic range of V/A values is where we generally observe the largest enhancements. The cell geometry pictures provide examples of how one can produce different V/A ratios along the x-axis relative to the central sphere. A maximum enhancement for each parameter set is reached at a value of V/A where lipids still outnumber proteins (shown on upper x axis). Increasing (a) protein-lipid affinities and (b) lipid concentrations produces greater possibilities for enhancement. (c) Increases in the membrane stickiness (K_a_^PM^[M]_eq_) produces monotonic increases in enhancement for all values of γ>1. (d) For lower expression levels relative to binding strength (K_a_^PP^ = 10^6^M^-1^), membrane localization can act as a switch to turn on assembly from <50% to >50% (shaded areas). (e) Timescales to equilibrate were calculated from simulations of both ODEs (solid lines) and reaction-diffusion (RD) (green points) (Methods). Weak lipid binding can reduce speeds (blue) relative to pure solution binding (dashed). The approximate theoretical bounds shown here for time-scales of binding either purely in solution (dashed) or on the membrane (gray) derive from the kinetics of irreversible association (S1 Text section 4C).

We derive an additional approximate equation for [M]_eq_ to provide a complete equilibrium theory of complex formation applicable to all experimental regimes, and we validate this equilibrium theory through extensive simulations of ordinary differential equations (ODEs) (Fig 2 and Fig 3, Methods). To briefly outline the derivation, we consider two limiting conditions for localization to the membrane: either there are no protein-protein interactions (K_a_^PP^ = 0), giving ${\left[M\right]}_{eq}^{0}$, or complete protein-protein complex formation (K_a_^PP^ = ∞), giving ${\left[M\right]}_{eq}^{Coop}$ (Fig 1D and 1E). These two bounds are indicated by dashed lines in Fig 2B and both limits are independent of K_a_^PP^. We can continuously interpolate between them (Fig 2B) using the definition

$${\left[M\right]}_{eq}={\left[M\right]}_{eq}^{0}\left(1-\lambda \right)+{\left[M\right]}_{eq}^{Coop}\lambda ,$$ (4)

where ${\left[M\right]}_{eq}^{0}$ is the root of a quadratic equation, ${\left[M\right]}_{eq}^{Coop}$ is the root of a cubic equation, and λ is the function of K_a_^PP^ (also the root of a quadratic) that smoothly interpolates between them (see Methods for detailed derivations). This theory then provides a complete description of the equilibrium concentrations of all species, as from K_a_^eff^, one can directly calculate the total complexes formed. The partitioning of complexes between solution and the membrane can subsequently be derived utilizing the equilibrium relations of Fig 1 (S1 Text section 2A). The larger the enhancement, the more complexes must be on the membrane (S1E and S1F Fig).

### Bounds on binding enhancement are determined by V/A and K_a_^PP^/K_a_^2D,PP^

Using our main result, Eq 3, one can predict when and by how much membrane binding will enhance complex formation of binding pairs without performing any simulation or experiment. Further, we can assess whether this enhanced complex formation is robust to perturbations in binding affinities or concentrations. To establish possible values for K_a_^eff^, we first ask: are there any cases where membrane binding will reduce protein complexation, i.e. K_a_^eff^<K_a_^PP^? To answer this, we consider the case where all proteins are on the membrane ($K_{a}^{P_{n}M}{\left[M\right]}_{0}\to \infty$), such that we have pure 2D binding and K_a_^eff^ = γK_a_^PP^. Reduced protein complexation will occur only if γ<1, or V/A<2σ. The size of σ controls the relative strength of K_a_^2D,PP^ vs. K_a_^PP^ and varies from one binding pair to another, but experiment and theory indicate it is of the nanometer length-scale [21, 22, 26]. We therefore collected the V/A ratios for a wide range of cell types and organelles to illustrate that in nearly all cells, V/A>2σ (~20nm) and thus membrane localization will enhance binding (S2 Fig). Indeed, targeting the plasma membrane in most cells results in γ values much greater than 1, in the range 10–1000 (S2 Fig). Ultimately, the V/(Aσ) ratio is absolutely central in controlling observed enhancement, as it sets the maximum achievable K_a_^eff^. In most cases proteins will end up mixed between solution and membrane, and from Eq 3 this gives us K_a_^PP^≤K_a_^eff^<γK_a_^PP^ (Fig 2). In the cases where only one protein binds to lipids, all 2D localization benefits are lost and no enhancement occurs: K_a_^eff^ = K_a_^PP^ (Fig 2E).

### Cooperativity emerges in protein-lipid binding

An important feature that our analysis captures is the coupling that emerges between protein-protein affinity and protein-lipid binding due to membrane localization. If a bound protein-protein complex localizes to the membrane by binding one lipid, binding to a second lipid then becomes a 2D rather than a 3D search [12] (Fig 1D and 1E). Thus, stabilization of proteins on the membrane is achieved not only through strong protein-lipid interactions, but by feedback from strong protein-protein interactions. This cooperative effect for lipid binding (binding of one lipid changes the affinity for the second lipid) produces the unexpected result that the number of proteins bound to the membrane is dependent on the protein-protein interaction strength (Fig 2B). To contrast, one could instead consider lipid binding as simply partitioning proteins between solution and membrane, after which they form complexes as in Fig 1A and 1C. This simplified interpretation, shown in gray dashed lines in Fig 2, does not capture the cooperative effect (Fig 1D and 1E) and is clearly wrong for strong binding proteins that rely on cooperativity to stabilize complex formation (see S1 Text section 2D for details).

### How binding enhancement responds to perturbations and factors external to the model

With our theory, it is possible to directly probe how changes in cell geometry, binding affinities, or concentrations will regulate enhancement. Although the model is too simple to fully describe multi-component assembly, by varying the input parameters to mimic competing cytosolic factors, one can evaluate the relative importance of concentration, affinities, and geometry. This is particularly true of equilibrium *in vitro* experiments. For changes to geometry, we first note that the equilibrium results depend only on the ratio and not the absolute size of V or A. The membrane does not have to surround the solution volume like the plasma membrane but can reflect binding to the outsides of liposomes, for example, which allows for studying any V/A ratio. Although it may seem that increasing V/A (through γ in Eq 3) always increases K_a_^eff^, this is not the case when [M]_0_ is kept constant in its natural units of μm^-2^ (it is then converted into Volume units by multiplication by A/V). V and A thus also control the initial copy numbers of proteins and lipids separately, such that large values of V/A have a great excess of proteins over lipids. This drives K_a_^eff^⟶K_a_^PP^ (Fig 3A and 3B). For most physiologic V/A values (~0.05–20μm S3 Table) however, and physiologic concentrations of proteins (1nM-10μM) or lipids (10^3^−10^5^μm^-2^ S4 Table), proteins are not in great excess, meaning significant enhancement is achievable depending on the membrane localization strength (Fig 3A and 3B).

#### Membrane composition

Because the membrane is described in our model only by its surface area A and the concentration of lipids targeted by the protein lipid-binding domains, the spatial and chemical heterogeneity of cell membranes is not captured. The stickiness of a membrane for specific proteins is captured in our model, however, by the dimensionless product K_a_^PM^[M]_eq_, which is simply equivalent to one protein’s ratio of membrane bound to unbound copies (e.g. [MP_1_]/[P_1_]) at equilibrium. As is clear from Fig 3C, the enhancement is a monotonically increasing function of K_a_^PM^[M]_eq_ for all γ>1 geometries. Hence with either stronger affinity for the membrane (K_a_^PM^) or a higher concentration of target lipids ([M]_0_), localization to the membrane and thus enhancement will increase. Fig 3C also illustrates how once K_a_^PM^[M]_eq_ exceeds values of ~10, maximum enhancement is reached and increasing the stickiness of the membrane does not change the resulting binding. In the S1 Text section 2B, we derive an explicit formula for this critical value of K_a_^PM^[M]_eq_, as well as the corresponding critical value for lipid concentration, [M]_c_ (S3A and S3B Fig). If the lipid concentration and affinity contribution to stickiness can be deconvoluted, as is the case for proteins that target individual lipids (such as many PI(4,5)P_2_ binders, Table 1) at essentially a 1:1 ratio, one can individually evaluate how decreases in either lipid concentration (Fig 3B) or affinity (Fig 3A) will lower enhancement. Importantly, we find that surprisingly low lipid concentrations are sufficient to drive maximal enhancement. For V/A = 1μm, (about the value for yeast cells), the PI(4,5)P_2_ concentration of 2.5x10^4^μm^-2^ produces close to the maximum in enhancement, meaning adding more lipids makes minimal difference (Fig 3B, S3A and S3B Fig). For proteins such as BAR domains, in contrast, assigning values for affinity and lipid concentration would require a composition dependent interpretation of these values, as BAR domains are less selective for individual lipid types and may only bind stably to clusters of lipids rather than 1:1 (see S1 Text section 5 for extended discussion). Nonetheless, any change in membrane composition that increases its stickiness towards any specific membrane binding domains will clearly drive up binding interactions between associated protein pairs (Fig 3C).

#### Competition for protein and lipid binding

Thus far we have said little about protein concentrations or K_a_^PP^, as enhancement is independent of their magnitude when lipids are in excess. However, these protein variables always determine when enhancement acts as a switch to turn on complex formation. Some proteins are perfectly capable of forming strong complexes in solution, whereas protein pairs with less than 50% complexes formed in solution can experience dramatic increases in bound complexes (Fig 3D, S3E Fig). The dependence of enhancement on protein concentration is also monotonic for fixed geometries: as protein concentration drops, enhancement increases (S3D Fig). Hence, competition for binding any of our protein binding pairs in solution would be expected to lower initial concentrations of each component. This will increase the ultimate enhancement (S3D Fig) and in many cases, make the proteins more sensitive to localization as a trigger for assembly (Fig 3D). If competition for protein binding partners involved other proteins that also bound to the membrane, then enhancement could be increased, but this extension beyond the Fig 1 model would have to be quantified via simulation. Competition for lipid pools, on the other hand, will always decrease enhancement, as shown in Fig 3B and explored further below for CME proteins.

#### Sensitivity to protein-protein affinity

Mutations to proteins would largely affect their affinities, either for their protein or their lipid partners. As noted above, however, even many-fold changes in affinity may have minimal consequences on measured enhancement (S3G Fig), and on resulting complex formation, due to the nonlinear dependence of complexes on affinity. For mutations to ENTH/ANTH domain containing proteins, a high concentration of target PI(4,5)P_2_ lipids means that most proteins bind to the membrane, where they will form maximal complexes, at moderate K_a_^PM^ values. Under these conditions, enhancement and total complexes will not change significantly even with 10-fold changes in K_a_^PM^[27] (S3H Fig). Decreases in K_a_^PP^ can have similar effects, either not affecting enhancement when membrane stickiness is already high, or otherwise increasing enhancement (S3C Fig, Fig 2). For complex formation, decreases in K_a_^PP^ can be much more significant, acting to increase the sensitivity of complex formation to localization, such that it is more likely to act as a trigger for assembly (S3E Fig). Mutations that asymmetrically affect the K_a_^2D^ values would result in changes in σ values, with smaller values always driving larger enhancement and stronger complexation on the membrane (S3F Fig).

### Timescales of assembly vary strongly with binding affinity

Our theory (Eq 3) only describes the equilibrium state of the model. However, we can determine speeds of assembly via simulation. Now, the binding rates and the absolute values of V and A (not just the ratio) will influence the kinetics (all simulation inputs in S2, S3, and S4 Datasets). For these time-scales, we find that protein-lipid affinities K_a_^PM^ are most often shown to be critical in controlling the overall time-scales of complexation, even driving slow-downs in speeds relative to solution binding (Fig 3E, S4 Fig). Changes in diffusion from solution to the membrane (about 100 times slower) affect the magnitude of association and dissociation rates and are captured implicitly in our ODE simulations (Methods), and explicitly in our spatially resolved reaction-diffusion simulations [19, 20]. However, the influence of diffusion on the reaction rates is rarely a dominant factor in physiological rate regimes (S4 Fig), indicating it is the binding strengths rather than slow 2D diffusion that determine assembly speeds. However, we note that our comparison of ODE and RD kinetics was performed in relatively small RD systems due to simulation costs, and it is true that as spatial dimensions increase, times to diffuse to reach the membrane will influence the overall equilibration times. The timescales we calculated for protein pairs and scaffold mediated systems (S4B and S4C Fig) were performed using ODEs at their corresponding cellular dimensions (S3 Table): V = 1200 μm^3^ (human) and V = 37.2 μm^3^ (yeast). Performing RD simulations at these dimensions would produce slower relaxation times, particularly for human cells, due to the time required to reach the surface. Crowding would also lower effective diffusion constants of proteins, although the decrease in time-scales to equilibrate would be negligible unless binding rates were strongly diffusion-influenced (Methods).

### Biological relevance for proteins in CME

**Fig 4:**
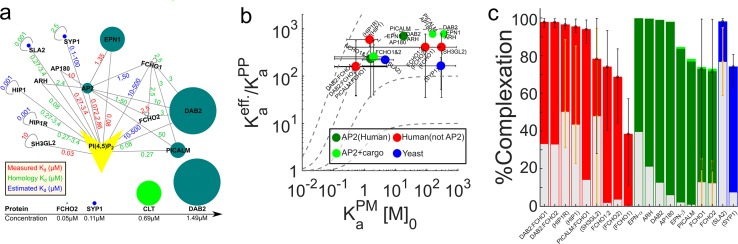
Membrane localization triggers strong complex formation for pairs of protein binding partners involved in clathrin-mediated endocytosis. (a) Interactions between lipid binding human (green) and yeast (blue) CME proteins are shown along with K_d_^PP^ values (μM) measured (red text), inferred through structural and functional homology (green), or estimated (blue) (Table 1, S1 Table, S3 Dataset). Sizes indicate concentrations (Table 1). (b) Enhancements for each of these CME binding pairs, following the model of Fig 1, were calculated using Eq 3 and verified through numerical simulation of ODEs. Pairs involving human AP-2 are in dark green, light green points involve AP-2 with cargo binding-adjusted K_a_^PM^, with red and blue points showing other human and yeast proteins, respectively. The x-axis is the average membrane stickiness for each pair, and here we assume σ = 1nm (see S5 Fig for σ = 10nm). For poorly characterized lipid binding affinities, we considered ranges of values (error bars in x), producing ranges of enhancements (error bars in y). Gray lines are guides for fixed V/A ratios. Protein names in parentheses are homo-dimers. (c) The percent of proteins in complexes increases from solution binding levels (gray bars) as a result of membrane localization (colored bars). All results and parameters used for all data points in S3 Dataset.

To test the biological relevance of membrane localization for driving complex formation and assembly, we collected biochemical (Table 1, S1 Table, S2 Table), concentration (Table 1, S4 Table), and cellular geometry data (S3 Table) for interactions among 37 membrane targeting proteins in yeast and human cells, including 22 proteins involved in clathrin-mediated endocytosis (CME). We first study only individual protein pairs that can bind according to our model of Fig 1, (S1 Table) shown in Fig 4A: the membrane binding proteins AP-2, DAB2, ARH, FCHo1, FCHo2, HIP1, HIP1R, PICALM, SH3GL2, EPN, AP180, SLA2, and SYP1. In Fig 4B and 4C we show results of binding between specific pairs. We used cytosplasmic concentrations of the proteins (Table 1) and the targeted lipids (S4 Table), and the relative Volume and Area from their respective cell types (S3 Table). Binding constants are collected from previous experimental studies (Table 1, S1 Table, S3 Dataset), and for 2D binding constants we test values of σ = 1nm (Fig 4) and 10nm (S5 Fig). Our results thus provide quantitative insight into how these pairs in isolation would use membrane localization at physiologic conditions to drive their protein-protein interactions. For some proteins, such as AP-2, solution $K_{a}^{PP}$ values have been measured with partners (Fig 4A, S1 Table), but further experiments indicate that the proteins undergo minimal binding in solution due to conformational regulation [1]. Despite this additional regulation, membrane localization will still increase complex formation relative to what is observed in solution (γ>1), so the effect is quantified here using the measured $K_{a}^{PP}$ value (S1 Table). Using our theory along with simulations for verification and time-scales, we find that affinities of these CME binding pairs can be enhanced 10–1000 fold by binding to membranes (Fig 4B, S5 Fig for results with σ = 10nm). With limited binding in solution for most pairs, membrane localization then triggers a dramatic increase in complex formation (Fig 4C, S5 Fig). The central adaptor protein AP-2 is responsible for many of these interactions, showing the capacity to trigger assembly with nearly all of its binding partners (Fig 4C, green bars). Even though we assume binding is possible for AP-2 in solution, it is still quite limited prior to localization. Not surprisingly, knockdown of AP-2 in mammalian cells causes severe disruption of endocytosis [28], underlining its secondary importance only to the irreplaceable clathrin [28] and PI(4,5)P_2_ [29]. We note that AP-2 can potentially bind up to three PI(4,5)P_2_ copies [30, 31], meaning that there will be less free lipids available for each AP-2. With fewer lipids, enhancement and complexation will be reduced, but is still quite large (S5 Fig). For some proteins such as FCHo1 (SYP1 in yeast), binding affinities (K_a_^PP^ or K_a_^PM^) are not available, and this F-BAR protein does not target a single lipid specifically. However, by considering ranges of membrane stickiness values, we can use our method to identify which combinations (S6 Fig) best describe the experimental observation that these proteins only localize effectively to membranes when they can bind other proteins [18, 32]. We find for this protein, membrane stickiness values of ~0.5 produce membrane targeting that is sensitive to protein-protein interactions, whereas once values exceed ~1, no partners are needed to target the membrane effectively (S6 Fig).

We further interrogate two additional mechanisms for stabilization at the membrane by lipid binding proteins such as AP-2, epsin, and Dab2 [3]. First, they each bind transmembrane cargo after membrane localization, which acts to effectively increase the K_a_^PM^ by increasing their residence time on the membrane. K_a_^PM^ is a factor of ~40 higher for AP-2 binding to PI(4,5)P_2_ when cargo is available [30]. Interestingly, these cargo stabilized interactions (Fig 4B, light green) do not make a significant impact on complexation when we assume the full 1% PI(4,5)P_2_ concentration is free to bind, as the numerous lipids outweigh a need for stronger binding (Fig 4C, light green). However, when we evaluate complexation with PI(4,5)P_2_ pools diminished by a factor of 10 due to assumed competition from other PI(4,5)P_2_ binders, now cargo stabilization via higher K_a_^PM^ does help recover strong complexation on the membrane (S5 Fig). This suggests that cargo binding, which is a main functional goal of CME, becomes a significant regulator of adaptor stabilization when competition from multiple adaptors limits PI(4,5)P_2_ binding. Second, when these adaptors can bind multiple partners with distinct appendage domains [33], we see more proteins on the membrane due to the increased difficulty of un-tethering from the membrane domain (S6B Fig).

In the cellular environment, CME proteins are of course not in isolation and can both compete and cooperate with one another to form higher order assemblies, induce conformational changes, and occupy lipid binding sites on the membrane. Thus we can only speculate about the role of localization in nucleating clathrin-coated pits *in vivo*. However, based on the above analysis showing that, physiologically, γ is greater than 1, membrane localization will drive clathrin towards complex formation. The initial nucleation of clathrin-coated pit sites is difficult to resolve experimentally because of the challenges in tracking the many participatory proteins simultaneously, and because prior to cage formation, the density of molecules is, by definition, low. Experiments have tracked the role of AP-2 and clathrin in nucleating sites [34], which we discuss below.

### Scaffold mediated interactions of CME proteins also exploit membrane localization

**Fig 5:**
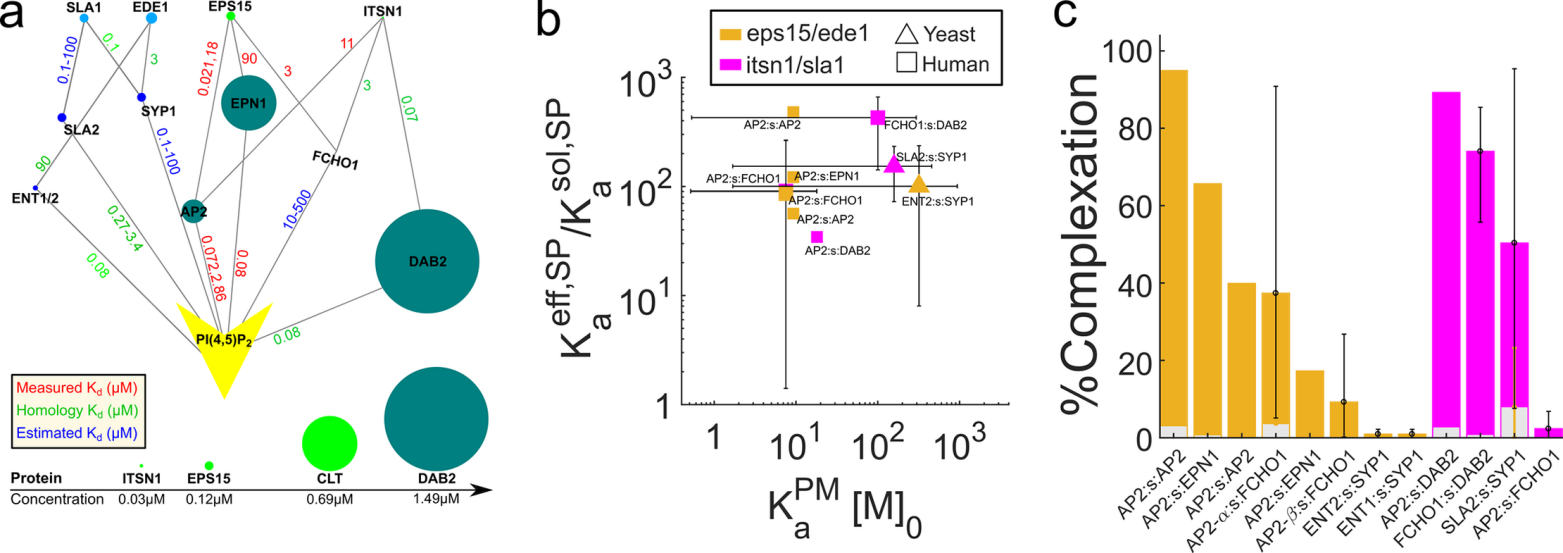
Scaffold-mediated interactions can also exploit membrane localization to drive complex formation in clathrin-mediated endocytosis. a) When sets of three proteins can form a complex and two of them also bind to lipids, localization is again capable of driving stronger complex formation. Yeast proteins in blue and human proteins in green, where the uppermost four (SLA1, EDE1, EPS15, ITSN1) are scaffold proteins that do not bind lipids. Affinities follow the same color scheme as Fig 4, and we have included only binding interactions between these proteins that are shown in parts b-c. (b) Because our primary model (Fig 1) no longer applies, we cannot use Eq 3 to predict enhancements, and instead use simulations of ODEs. The scaffold model is defined in S1 Text section 1C and S7 Fig, along with the definitions of K_a_^eff,SP^ and K_a_^sol,SP^, where neither is a true equilibrium constant and the equilibrium results of the simulations are used to calculate their defined values. Without membrane localization, K_a_^eff,SP^→K_a_^sol,SP^. Scaffold proteins (*s* in the labels) are eps15/ede1 (orange), or itsn1/sla1 (pink). (c) Complexation involves all three proteins (see S1 Text section 1D) and here again, the percent of proteins in complexes increases from solution binding levels (gray bars) as a result of membrane localization (colored bars). All results and parameters in S4 Dataset.

To go beyond our Fig 1 model of pairwise protein binding and thus characterize how scaffold proteins (Table 1: ITSN1, EPS15, EDE1 and SLA2) stabilize complex formation at the membrane despite not directly interacting with the lipids (model in S7 Fig, list of interactions in S2 Table), we simulate systems of ODEs, as Eq 3 no longer applies (Methods). We thus simulate interactions involving three proteins, two of which can bind lipids but not each other, and the third that binds both peripheral membrane proteins but not the membrane (Fig 5A). Our results in Fig 5B and 5C show that while scaffold mediated complexes can still capitalize on 2D localization for binding (Fig 5B), because localization is now mediated by peripheral membrane proteins that are at much lower concentrations than the lipid recruiters, we find that the increase in complex formation is less robust (Fig 5C), and is limited by concentration of the scaffold protein (S8A Fig).

### Clathrin cage nucleation and BAR domain oligomerization can exploit membrane localization

Thus far we have not discussed clathrin, the central component of the CME vesicles that does not actually bind to lipids itself. *In vitro* experiments find that clathrin polymerization on the membrane (via adaptor binding) is more robust than occurs in solution (with adaptors still present), supporting a role for membrane localization in its nucleation and assembly [2]. Clathrin is a trimeric protein with three binding sites to target peripheral membrane proteins. It polymerizes with itself into hexagonal lattices without competition from the peripheral membrane proteins. Thus, its interactions with peripheral membrane proteins not only increase the quantity of protein bound to the membrane, it can help drive 2D polymerization between clathrin trimers. Through (non-spatial) stochastic simulations (Methods) set-up to mimic recent *in vitro* experiments [1], we explored a range of clathrin-clathrin interaction strengths to show how membrane recruitment by the AP-2 adaptor [1] can enhance clathrin polymerization yield (S8 Fig). Although these simulations lack molecular structure or spatial resolution, they can track formation of multi-protein complexes and the important role of affinity and concentration in controlling these complexes. We find that clathrin localizes to the membrane first via AP-2 binding before assembling into cages in 2D for the most reasonable K_d_s of 10–100μM [35]. This result is supported by evidence from *in vivo* experiments that probe the early stages in the nucleation of clathrin coated pit sites through tracking of AP-2 and clathrin [34]. They found that clathrin arrives at the membrane most frequently (75%) as a single trimer, and bound to at least one but most often two AP-2 molecules [34]. Nucleation can then initiate in two ways: (A) another clathrin trimer localizes to the membrane via AP-2 and these trimers dimerize in 2D or (B) another clathrin trimer is directly recruited by the clathrin on the surface. Although the subsequent clathrin dimerization events were not resolved experimentally, preventing definitive evidence of membrane localized clathrin-clathrin assembly, the fact that each clathrin is bound to AP-2 suggests that AP-2 binding of clathrin is a prerequisite for initial clathrin dimerization. From our simulations, the (A) nucleation process is markedly dominant. There is a strong driving force both from affinity and from concentration for AP-2 to bind any of the 25000 PI(4,5)P_2_/um^2^, and correspondingly minimal drive for a solution clathrin to bind a small number of clathrin trimers localized to the surface. We note that because clathrin also arrives at the membrane as dimers or higher order complexes 25% of the time, solution binding of clathrin also contributes to nucleation of pit sites, but to a much lower extent [34]. Interestingly, once pit sites have formed, assembled clathrin cages exchange with solution clathrin with the aid of ATP-consuming proteins that facilitate remodeling of the clathrin cage [36]. Thus clathrin-clathrin interactions from solution certainly play an important role in the cell in maturing the pit sites [36].

CME proteins with BAR domains that dimerize, appear to oligomerize only on the membrane, and are functionally important for driving membrane deformation [17, 18] can also exploit localization to drive their binding interactions. We study isolated FCHO1/2 oligomerization and endophilin (SH3GL2) oligomerization, again using non-spatial stochastic simulations (S9 Fig). Here again we consider a range of K_a_^PM^ values to capture uncertainty in the membrane stickiness of these domains. We find the stoichiometry of the dimerization pair (homo or hetero) is central in determining whether large oligomers form. With matched pairs, homodimers such as endophilin form larger oligomers that feedback into higher stabilization at the membrane, whereas the disparity in FCHo1 and FCHo2 concentration (S1 Table) produces more isolated dimers. Experiments have shown that BAR domains exhibit stronger binding to curved membranes [17]. Because we lack this cooperative feedback in our model between oligomers tubulating membranes and thus potentially increasing affinity for subsequent proteins, our result can be interpreted as a lower bound on observed oligomerization.

In all cases, an important outcome of these strong binding interactions on the membrane is that they are difficult to disassemble, consistent with findings that unproductive assembly events observed *in vivo* [37] require the ATP-driven uncoating machinery for disassembly [2, 38]. Our results demonstrate that establishing the physiologic significance of these polymerization observations depends not only on protein concentrations and solution conditions, but also the V/A ratio. Thus, this ratio should be regarded as a critical factor in designing *in vitro* experiments to better reflect *in vivo* behavior.

### Biological relevance for membrane remodeling pathways in yeast

Lastly, our analysis motivates why diverse proteins that target membranes in yeast can follow pathways to assembly both similar and distinct from the CME proteins. In particular, the CME protein pairs produce limited protein-protein complexes when isolated in solution, but experience large enhancements due to membrane localization, triggering widespread protein-protein interactions only after binding to the membrane (Fig 4). For 15 yeast proteins involved in daughter cell budding, lipid regulation, and morphogenesis, we studied their pairwise binding in using cytoplasmic (yeast) concentrations (Table 1), lipid concentrations (S4 Table), cytoplasmic V/A ratios (S3 Table) and experimentally measured protein-lipid affinities (Table 1). We find binding enhancements are high (100–1000), similar to the CME proteins, indicating that binding will be promoted once proteins are on the membrane (S10 Fig, S2 Dataset). Although enhancements were readily measured for these yeast binding pairs because they were independent of K_a_^PP^ values (S2 Dataset), we could not directly compare complexation for these interactions as we did for the CME interactions because they lacked any K_a_^PP^ data. For binding enhancements, we found an exception in the coat forming proteins targeting endosomes (VPS5, VPS17, SNX4, SNX41), which only exhibit enhancements <20. These proteins target the PI(3)P lipid but most bind only weakly (K_d_^PM^>100μM) [39], limiting their enhancements despite a favorable V/A ratio at the endosome (S10 Fig, S3 Table). Unlike in CME, however, these coat proteins form stable interactions in solution [40]. Thus, rather than membrane binding triggering protein interactions, we would first expect the reverse: strong protein interactions in solution function to target and stabilize protein at the membrane through the cooperative effect (Fig 1D and 1E, Fig 2B). We test how the binding of the retromer components VPS5 and VPS17 to the endosome will be significantly enhanced by forming a higher order assembly in solution with the strong lipid binding cargo adaptor, SNX3 (S9 Fig). SNX3 targets PI(3)P with stronger affinity (~2μM) than either VPS5 or VPS17 [39], and is known to improve recruitment of the retromer to endosomes [40]. Once these small pre-assembled coat subunits are on the membrane, they can then continue to exploit localization to form larger protein coats.

### Conclusions

We conclude by noting that assembly on membranes is regulated to occur at specific times or sub-cellular locales, and our theory provides a useful aid in predicting the changes in local protein, lipid concentrations, and affinities that are necessary to trigger (or prevent) such assembly. Ultimately, our theory is most powerfully applied to interpreting *in vitro* results, due to the simplifying assumptions of the model, and can improve the design and quantitative interpretation of assays probing multi-protein complexation at membrane surfaces. Also, given known protein-protein and protein-lipid binding affinities, our theory can quantitatively predict the results of *in vitro* experiments that mimic Fig 1, thus avoiding the need for such measurements. Our results indicate that even relatively low lipid concentrations (i.e. PI(4,5)P_2_ at ~1% of plasma membrane lipids) can be sufficient in many cases to stabilize proteins to membranes and drive protein-protein interactions. We found that additional factors, such as cargo binding by adaptor proteins in CME, are only strong regulators of membrane localization or protein interactions under specific conditions. Since cargo-binding is known to influence the success of vesicle formation *in vivo*[41, 42], this suggests that the condition where total PI(4,5)P_2_ concentration is reduced to mimic competition from other proteins is more physiologically relevant. A fruitful means of exploring in more detail the role of cytoplasmic factors, as well as spatial heterogeneity, crowding, and non-equilibrium dynamics, is through reaction-diffusion simulations, although we note the results will then be dependent on many additional parameters. Overall, the theory we provide here offers a general and useful quantitative guide for predicting when or if membrane localization plays a role in the cellular control of self-assembly.

## Methods

### Theoretical derivations

#### Derivation details of K_a_^eff^ (Eq 3)

The exact solution (both equilibrium and time-dependence) to our model of proteins interacting and recruiting to membranes (Fig 1) can only be obtained numerically. Starting from our definition in Eq (1), ($K_{a}^{eff}=\frac{\left({\left[P_{1}P_{2}\right]}_{eq}+{\left[MP_{1}P_{2}\right]}_{eq}+{\left[P_{1}P_{2}M\right]}_{eq}+{\left[MP_{1}P_{2}M\right]}_{eq}\right)}{\left({\left[P_{1}\right]}_{eq}+{\left[MP_{1}\right]}_{eq})({\left[P_{2}\right]}_{eq}+{\left[P_{2}M\right]}_{eq}\right)}$), we input pairwise equilibrium expressions for each species in the numerator, where the complete list of pairwise equilibria illustrated in Fig 1 are given by equations:

$$P_{1}+P_{2}⇋P_{1}P_{2}\;(K_{a}^{PP})$$ 5.1

$$M+P_{1}⇋{\mathrm{MP}}_{1}\;(K_{a}^{P_{1}M})$$ 5.2

$$M+P_{2}⇋P_{2}M\;(K_{a}^{P_{2}M})$$ 5.3

$$M+P_{1}P_{2}⇋{\mathrm{MP}}_{1}P_{2}\;(K_{a}^{P_{1}M})$$ 5.4

$$P_{1}P_{2}+M⇋P_{1}P_{2}M\;(K_{a}^{P_{2}M})$$ 5.5

$${\mathrm{MP}}_{1}+P_{2}⇋{\mathrm{MP}}_{1}P_{2}\;(K_{a}^{PP})$$ 5.6

$$P_{1}+P_{2}M⇋P_{1}P_{2}M\;(K_{a}^{PP})$$ 5.7

$${\mathrm{MP}}_{1}+P_{2}M⇋{\mathrm{MP}}_{1}P_{2}M\;(K_{a}^{PP}/(2\sigma^{PP}))$$ 5.8

$$M+P_{1}P_{2}M⇋{\mathrm{MP}}_{1}P_{2}M\;(K_{a}^{P_{1}M}/(2\sigma^{P_{1}M}))$$ 5.9

$${\mathrm{MP}}_{1}P_{2}+M⇋{\mathrm{MP}}_{1}P_{2}M\;(K_{a}^{P_{2}M}/(2\sigma^{P_{2}M}))$$ 5.10

where 5.8, 5.9 and 5.10 are all in 2D. Reactions in 2D list the 2D K_a_ values and thus require species be in units of Area^-1^. To solve all species in consistent units, where we will use solution concentrations with units V^-1^, the listed 2D K_a_ values must be multiplied by V/A. This is the origin of the *γ* factor, $\gamma =\frac{V}{2A\sigma }$. We note that the protein-protein interactions have the same equilibrium constant when one or both proteins are in solution (Eqs 5.1, 5.6 and 5.7). This definition preserves detailed balance, and thus an equilibrium steady-state. This is based on the assumption that because the binding equilibrium is still driven by the solution exchange of (one) protein with the complex using the same interfaces, the relative fraction of bound and unbound states is the same.

Inputting Eqs 5.1, 5.6, 5.7 and 5.8 into the numerator of Eq 1 and dividing numerator and denominator by the factor [P_1_]_eq_[P_2_]_eq_ gives:

$$K_{a}^{eff}=K_{a}^{PP}\left(\gamma \frac{{\left[MP_{2}\right]}_{eq}{\left[MP_{1}\right]}_{eq}}{{\left[P_{2}\right]}_{eq}{\left[P_{1}\right]}_{eq}}+1+\left(\frac{{\left[MP_{1}\right]}_{eq}}{{\left[P_{1}\right]}_{eq}}+\frac{{\left[MP_{2}\right]}_{eq}}{{\left[P_{2}\right]}_{eq}}\right)\right)/\left(\left(1+\frac{{\left[MP_{1}\right]}_{eq}}{{\left[P_{1}\right]}_{eq}}\right)\left(1+\frac{{\left[MP_{2}\right]}_{eq}}{{\left[P_{2}\right]}_{eq}}\right)\right)$$ (6)

And finally using Eqs 5.2 and 5.3 above (Eq 2 of the main text), we recover our main result, Eq 3. We also note if P_1_ and P_2_ target distinct lipids, the [M] concentrations will be subscripted accordingly. Thus Eq 3 of the main text is exact. However, a separate equation for [M]_eq_ is needed that will be approximate.

#### Derivation details of [M]_eq_ (Eq 4)

Our equation for the unbound lipids at equilibrium, [M]_eq_, is a function of two limiting cases for protein localization to the membrane with a smooth interpolation in between defined via Eq 4 of the main text (${\left[M\right]}_{eq}={\left[M\right]}_{eq}^{0}(1-\lambda )+{\left[M\right]}_{eq}^{Coop}\lambda$). In the first extreme (K_a_^PP^ = 0), we solve for unbound lipids at equilibrium based solely on protein-lipid interactions, M+P⇋MP, giving the familiar quadratic root

$${\left[M\right]}_{eq}^{0}=\frac{1}{2}\left({\left[M\right]}_{0}-{\left[P_{tot}\right]}_{0}-\frac{1}{K_{a}^{PM,av}}+\sqrt{{\left({\left[M\right]}_{0}-{\left[P_{tot}\right]}_{0}-\frac{1}{K_{a}^{PM,av}}\right)}^{2}+4{\left[M\right]}_{0}/K_{a}^{PM,av}}\right)$$ (7)

This equation recovers [M]_eq_~[M]_0_, the initial concentration of lipids, when [M]_0_>>[P_tot_]_0_ = [P_1_]_0_+[P_2_]_0_. The K_a_^PM,av^ is the average from both proteins. For significant differences between affinities and protein populations, the weighted average is most accurate, $K_{a}{}^{PM,av}=(K_{a}^{P_{1}M}[P_{1}]+K_{a}^{P_{2}M}[P_{2}])/([P_{1}]+[P_{2}])$, where using [P_1_]_eq_’s rather than [P_1_]_0_ is more accurate. For the second extreme, (K_a_^PP^ = ∞) we now treat all proteins as bound in complex, creating an equilibrium between proteins with two lipid binding sites and the membrane. This gives us ${\left[M\right]}_{eq}^{Coop}$, which has a cubic root analytical solution (see S1 Text section 2C). Lastly, we interpolate between these two extremes (both independent of K_a_^PP^), using the function λ. The λ function is the fraction of proteins that are bound to one another (based on P_1_+P_2_⇋P_1_P_2_ with K_a_^eff^) out of the maximum possible,

$$\lambda =\frac{1}{2}\left({\left[P_{1}\right]}_{0}+{\left[P_{2}\right]}_{0}+K_{a}^{eff^{-1}}-\sqrt{{\left({\left[P_{1}\right]}_{0}+{\left[P_{2}\right]}_{0}+K_{a}^{eff^{-1}}\right)}^{2}-4{\left[P_{1}\right]}_{0}{\left[P_{2}\right]}_{0}}\right)/\min\left({\left[P_{1}\right]}_{0},{\left[P_{2}\right]}_{0}\right)$$ (8)

These three results thus complete the equilibrium theory for Eqs 3 and 4. The function λ depends on K_a_^eff^, which can be calculated based on plugging ${\left[M\right]}_{eq}^{0}$ into Eq 3. All our theoretical results shown are based on this definition. However, we note that the final value of K_a_^eff^ dependent on λ can also be then fed back into this Eq 8 to self-consistently converge to an improved result for K_a_^eff^.

An important feature of Eq 4 is that it produces the correct limiting behavior as λ goes from 0 to 1. We emphasize that although the equation for ${\left[M\right]}_{eq}^{Coop}$ is cumbersome, simply setting λ = 0 will already give very good accuracy in reproducing the exact result, with noticeable errors only expected when both the lipid concentration is small relative to the total proteins and the K_a_^PP^ is large. We note that the relative error in the full Eq 4 is quite small, although for large V/A ratios (larger than those observed physiologically), the error grows and produces overestimates of theoretical enhancement ratios relative to the numerical solution (Fig 3A and 3B). We provide Matlab code (solveKaeff.m) that performs this complete calculation for any model.

### Definition of microscopic and macroscopic rates for simulation

To simulate the systems of ordinary differential equations (ODEs) for Fig 1 (S1E Fig, S1A Text section), we need macroscopic rates, and to simulate the single-particle reaction-diffusion system (RD), we need microscopic rates (also known as intrinsic rates in the Smoluchowski theory [43]) in both 3D and 2D. The macroscopic rates emerge based on the dynamics of the more detailed microscopic system, and can therefore be constructed to optimally match the kinetics of the ODE simulations to the RD simulations. We note that these definitions are specific to the kinetics, as the equilibrium of both simulation approaches will be identical due to their matching equilibrium constants.

The ODE simulations do not account for space or explicit diffusion. Here, we define their rates to implicitly account for changes to diffusion and thus best match the RD simulation kinetics. That way, discrepancies between kinetics of ODE and RD results can be attributed to explicit spatial heterogeneity influencing the binding interactions. Macroscopic association (on-) rates can be defined in 3D from the intrinsic rate of the Smoluchowski model via the relation [44]:

$$k_{on}^{3D}={\left(\frac{1}{k_{a}^{3D}}+\frac{1}{4\pi \sigma D_{tot}^{3D}}\right)}^{-1},$$ (9)

where k_a_ is the intrinsic association rate that captures the barrier to complex formation for species in contact at binding radius σ, and D_tot_ is the sum of both species’ diffusion constants. The macroscopic off-rate can be defined in all dimensions via

$$k_{off}=k_{on}/K_{a}.$$ (10)

The intrinsic dissociation rate k_b_ is defined via the corresponding equation, k_b_ = k_a_/K_a_, with all off-rates having the same units in all dimensions of s^-1^. In 2D, there is no single macroscopic rate constant independent of the system size or concentrations [20]. However, one can define a macroscopic 2D rate, built on theory from Szabo et al [45], that provides optimal agreement with the corresponding spatial reaction-diffusion simulations via [20]:

$$k_{on}^{2D}={\left(\frac{1}{k_{a}^{2D}}+\frac{1}{8\pi D_{tot}^{2D}}\left[\frac{4\log\left(b\left(\rho \right)/\sigma \right)}{{\left(1-\sigma^{2}/b{\left(\rho \right)}^{2}\right)}^{2}}-\frac{2}{\left(1-\sigma^{2}/b{\left(\rho \right)}^{2}\right)}-1\right]\right)}^{-1}$$ (11)

where

$$b\left(\rho \right)=2\sqrt{A/\left(\pi \;\max(N_{P_{1}},N_{P_{2}}\right)+\sigma^{2})}$$ (12)

is a length scale that is defined based on the more concentrated of the reacting species P_1_ or P_2_ in the surface area *A*.

The important interpretation of Eqs 9 and 11 is that, unless k_a_ is large, even substantial (factor of 10 or more) changes to the diffusion constant will have a relatively small impact on the macroscopic rate. It is not until macroscopic rates reach values of ~10^6^-10^7^M^-1^s^-1^ that they become strongly diffusion influenced and thus sensitive to changes in diffusion.

Our 2D intrinsic rates are defined relative to our 3D rates via

$$k_{a}{}^{2D}=k_{a}{}^{3D}/(2\sigma ),$$ (13)

and unbinding rates

$$k_{b}{}^{2D}=k_{b}{}^{3D},$$ (14)

which produces the equilibrium relation defined in the main text, K_a_^2D^ = K_a_^3D^/(2σ). We assume here that the dissociation rates are the same from 3D to 2D. It is the association rates that capture two species finding one another in a specific spatial dimension. This definition of Eq 13 also can be shown to preserve the reactivity of the binding interaction in the Smoluchowski model from 3D to 2D, independent of changes to diffusion (S1 Text section 4A). For the macroscopic 2D rates, k_on_^2D^, we used Eq 13 in Eq 11, which allows us to capture effects of diffusion towards timescales of binding in k_on_^2D^, as D^2D^ is ~100 times lower than D^3D^. Transitioning from solution to the membrane via binding lipid or protein involves a 3D search, and thus uses the corresponding 3D rates. See S1 Text section 3B for further discussion.

Ultimately, the results of K_a_^eff^ are only sensitive to equilibrium constants such as K_a_^2D^ and therefore the size of σ, rather than sizes of relative rates. This length scale σ encodes thermodynamic properties of the molecules involved in the binding reaction and is of the nanometer range [22]. In general, the value of σ therefore depends on the proteins involved, but σ (or K_a_^2D^), is almost never measured. We extract σ~7nm (from V/A = 6.7μm and K_a_^eff^/K_a_^PP^≈500) in the experimental measurement of 2D binding between calmodulin and a target peptide [21]. Smaller σ values have been observed [26]. For simulations, we thus used either 1 or 10nm. We used the same value for the protein-protein (*σ*^*PP*^) or protein-lipid ($\sigma^{P_{1}M}$, $\sigma^{P_{2}M}$) 2D binding interactions, although only *σ*^*PP*^ appears in Eq 3. The size of these values is constrained to ensure an equilibrium steady-state is reached, and the simplest solution has that $\sigma^{PP}=\sigma^{P_{1}M}=\sigma^{P_{2}M}$.

### Computer simulation methods

#### Numerical solutions of ODEs

The majority of our simulation results (exceptions noted below) come from numerically solving the system of ODEs describing the change in time of the concentrations of all protein, lipid, and bound species (S1 Fig) via Mathematica (Equations listed in S1 Text section 1A). The initial conditions had all proteins and lipids unbound and all proteins in solution. For all simulations, our default was k_off_ rates of 1s^-1^. Then k_on_^3D^ was defined via Eq 10. Exceptions were for proteins with known rates, and for the few simulations where to prevent k_on_^3D^ from exceeding the diffusion-limited value of 4πσD_tot_, we used k_off_ = 4πσD_tot_/K_a_^PP^. Although the ODEs do not use diffusion constants, we did need them to define k_a_^3D^ (Eq 9), then k_a_^2D^ (Eq 13), then k_on_^2D^ (Eq 11). We used D^3D^ = 50μm^2^/s and D^2D^ = 0.5μm^2^/s for each species, both reasonable estimates for diffusion in solution and lipid diffusion[12] [23]. For equilibrium measurements (Fig 2 and Fig 3A and 3B) we also simply defined k_on_^2D^ = k_on_^3D^/(2σ). To calculate the percentage of proteins in complex, we used $\%\;Complexation=100*\frac{{\left[Complex\right]}_{eq}}{min({\left[P_{1}\right]}_{0},{\left[P_{2}\right]}_{0})}$.

#### Simulations with scaffold proteins

For the scaffold-mediated system (S7 Fig), the addition of the scaffold protein (SP) with two binding sites, one for each peripheral membrane protein, meant a total of 14 species could be formed, producing a larger system of ODEs to solve. The ODEs were solved with Mathematica. Both K_a_^eff,SP^ and K_a_^sol,SP^ were extracted from simulations for all systems (S1 Text sections 1C and 1D, S2 Table), with and without membrane present, respectively. This allowed us to measure the enhancement in binding due to localization, just as for the pairs, even though here K_a_ is not a true equilibrium constant for complex formation.

#### Rule-based stochastic simulations of higher order oligomers and clathrin lattice formation

To study not only dimerization or binding mediated by a single scaffold protein, but binding of components into chain-forming oligomers or clathrin lattices, we performed Gillespie simulations[46] written in our lab using a rule-based implementation. Rule-based implementations[47] allow one to track formation of large multi-protein complexes including dimers, trimers, n-order oligomers, etc., without having to enumerate all possible complexes in advance, which is a huge challenge to encode in a system of ODEs. These simulations lack spatial or structural detail, so although we can track complexes formed, we cannot visualize assemblies or structural features. Nonetheless, the results correctly capture how binding rates, concentrations, and membrane localization control complex formation, and thus are a useful initial model approach to quantifying the role of dimensionality reduction. To study BAR domain proteins forming oligomers, the BAR proteins each contained their dimer forming interaction sites as well as an additional non-competing site, allowing oligomeric filaments to form. Because oligomerization was not observed in solution even at ~100μM concentrations [11], we assume weak oligomer contacts of 500μM, which will produce <5% of proteins in higher order complexes in solution. We calculate %oligomerization as the number of bound oligomer sites on the partner at lower concentration, relative to its total concentration. Full simulation conditions are in S4 Dataset.

To study clathrin polymerization, each trimer leg (one clathrin molecule has three trimer legs) was able to bind to any trimer leg of another clathrin molecule, and these interactions did not compete with adaptor binding. The ability of clathrin to interact with other trimers was assumed to be independent of its interactions with the adaptor AP-2 and no cooperative binding of clathrin was included, to minimize the number of adjustable parameters and consider the simplest model of cage formation (S2 Table, S8 Fig). Clathrin polymerization was simulated for the *in vitro* experimental conditions reported in Kelly et al [1]. We extracted a V/A ratio of 9.46μm and a lipid concentration of 54,668 μm^-2^ from the study.

#### Spatially resolved reaction-diffusion simulation details

Single particle reaction-diffusion (RD) simulations were used to measure time-scales of assembly formation (Fig 3E, S4 Fig) in a way that explicitly captured the spatial distribution of proteins and lipids and the diffusion of species to contact. We used the Free Propagator Reweighting (FPR) algorithm, an efficient and highly accurate method for studying reactions between diffusing species at spatial and single molecule resolution both in solution [19] and on the membrane [20]. All lipids are initialized in the membrane plane, which is the bottom plane of the simulation box, distributed randomly. Each protein is a sphere, and binding to a lipid (also a sphere) does not prevent binding to the protein partner, and vice versa. The simulation box has periodic boundaries in the x and y dimensions, and the z dimensions are both reflective, with the lower z plane containing the reactive lipids. The equilibrium properties of the RD simulations agreed with the ODE simulations, because of the conserved equilibrium constants (S4 Fig). The time-dependent properties of the RD simulations did not differ significantly from the ODEs (Fig 3E, S4 Fig) due firstly because we took care in assigning corresponding macroscopic and microscopic rate constants above. Secondly, the spatial dimensions of the RD systems we simulated were small enough (box of 0.47x0.47x0.76μm) that diffusion to reach the membrane did not slow down equilibration. For box sizes with larger distances to reach the membrane, however, the RD equilibration time slows relative to the ODEs due to this spatial effect.

The FPR code for performing these RD simulations is available for download from github.com/mjohn218/FPR_simulator.

### Collecting biochemical data, *in vivo* geometry, and concentrations

In Table 1 we list all the human and yeast proteins for which we were able to collect sufficient biochemical data on lipid and protein interactions. The 20 lipid-binding yeast proteins were retained from a larger list of 139 peripheral membrane proteins (PMP) identified from the Uniprot database as having lipid binding activity in yeast (S1 Dataset). Between this set of 139 PMPs, we found 396 interactions via BioGRID, however, only 17 pairs (S1 Table) involved partners with known K_a_^PM’^s. The 15 human proteins studied are all involved in CME and their biochemical data (Table 1, S1 Table, S3 and S4 Datasets) was collected via extensive literature curation. To study scaffold-mediated interactions (S2 Table), we identified all possible interactions that involved a non-membrane binding protein that could simultaneously and non-competitively bind to two of our PMPs. For the yeast proteins, these interactions could be identified from the manually curated interface interaction network for CME proteins [48]. There was a relatively small number of examples where a single scaffold protein was capable of bridging two PMPs (S2 Table). These interactions in humans/yeast involved clathrin/clathrin, eps15/ede1, or itsn1/sla1.

In S3 Table we collected volume and surface areas for cells and organelles with justifications provided. Because the cytoplasmic volume typically constitutes 50–60% of the total cell volume in mammalian cells, our V/A ratios set the solution volume as 60% of the total cell volume for all cell types. Lipid concentrations are collected in S4 Table. The concentrations of specific lipids on specific membranes have only been quantified in a few cases, such as PI(4,5)P_2_ having an average concentration of 2.5x10^4^μm^-2^ on the plasma membrane in mouse fibroblasts [15]. We used this concentration as a gold standard, due to its relative consistency across measurements [13, 15], and other phosphoinositide concentrations were quantified relative to this one. We curated literature to collect the necessary copy numbers of each lipid in the cell, and their distributions across organelles. Lastly, protein concentrations were defined from copy numbers measured in yeast [49] and human cells [50] (Table 1).

## Supporting information

S1 Text Sections 1–5: Extended methods and model descriptions. (PDF)

S1 Matlab program Takes user inputs on system parameters and calculates K_a_^eff^ and equilibrium concentrations of all species. (M)

S1 Table Pairwise protein-protein interactions (PPIs) and affinities. (PDF)

S2 Table Scaffold-mediated PPIs and higher-order assemblies, with affinities. (PDF)

S3 Table Volume and membrane surface area estimates for different cells and organelles. (PDF)

S4 Table Phosphoinositide (PtdInsP_n_) and phosphatidylserine concentrations across various organelles in a mammalian and a yeast cell. (PDF)

S1 Dataset Lipid binding affinities and literature for yeast peripheral membrane proteins. (XLSX)

S2 Dataset Protein-protein interaction pairs studied here for yeast. All simulation inputs and literature references. (XLSX)

S3 Dataset Protein-protein interaction pairs studied here for clathrin-mediated endocytosis. All simulation inputs and literature references. (XLSX)

S4 Dataset Scaffold-mediated and oligomer forming interactions studied here. All simulation inputs and literature references. (XLSX)

S1 FigThe simple theory developed here quantifies bound protein complexes for all systems with great accuracy.a,b) The relative error of the theoretically predicted K_a_^eff^ values compared with the exact numerical result from simulation, with data corresponding to the results of Fig 2A_1_ and 2C_1_, respectively. As expected, the error (~10^−8^) is negligible in (a) under the conditions of excess lipids simulated in Fig 2A_1_, as [M]_eq_ is nearly exactly predicted by our approximate theory. In (b), the error increases now that lipids are outnumbered. Error is highest for moderate K_a_^PP^ values, because here the predicted value of [M]_eq_ is farthest from either of the limiting (and exact) predictions of ${\left[M\right]}_{eq}^{0}$ or ${\left[M\right]}_{eq}^{Coop}$. The error reduces to values of 10^−3^ and 10^−2^ near these limits. c,d) From Eq 3 K_a_^eff^, we can directly calculate the concentration of bound protein-protein complexes, as K_a_^eff^ = [Complex]_eq_/(([P_1_]_0_-[Complex]_eq_)([P_2_]_0_-[Complex]_eq_)). Simulation (red) vs theory (black). c) V/A = 0.76, K_a_^PM^ = 10^4^M^-1^, [P_1_]_0_ = [P_2_]_0_ = 0.1 *μM*, [M]_0_ = 2.510^4^ *μm*^−2^. (d) Same as (c) except [P_1_]_0_ = [P_2_]_0_ = 2 *μM* and [M]_0_ = 10^3^ *μm*^−2^ e,f) The fraction of these complexes that are specifically on the membrane (Eq. S7). g) Network of reactions between all states. States with black outline all contain protein-protein complexes. Reactions in 2D are in green text. Protein-protein binding is otherwise in navy text, and protein-lipid binding in orange text.(TIF)

S2 Fig Membrane localization of proteins in a variety of cell types will produce increased protein binding interactions. a) Proteins in the cytosol can localize to membranes by binding specific lipids (yellow). Peripheral membrane proteins that do not bind directly can be bridged by a scaffold protein (green/gray) b) We collected the solution volumes (V) and membrane surface areas (A) for both plasma and organellar membranes in a variety of cell types (S3 Table). Only when the V/A ratio drops below 2σ, where here σ is set to 10nm, does the membrane reduce binding relative to solution (bottom black line). The only case found here is for proteins inside the yeast Mitochondria, which has a small volume but a large surface area due to the highly invaginated structure of the membrane. The V/A ratio for a sphere (V/A = R/3) is shown for reference in the diagonal black line.
(TIF)

S3 Fig Role of protein concentration, K_a_^PP^, mutations, and lipid concentration in enhancement and complex formation. a) Once the enhancement due to membrane localization is near to the maximum value, the addition of more lipids changes the binding equilibrium imperceptibly. Even a relatively low concentration of lipids is needed to trigger the maximum binding interactions, particularly with strong K_a_^PM^. b) This critical lipid concentration, [M]_c_, beyond which no further changes are observed in binding is derived in S1 Text (simulation results are points, theory is lines). We define maximum binding as within ε of K_a_^eff^ = γK_a_^PP^, with results here shown for ε = 0.01. c) Protein interactions between proteins with weak solution binding (low K_a_^PP^) or d) low protein concentrations benefit more widely from recruitment. This is because these systems will form minimal complexes in solution (with (K_a_^PP^)^-1^>[P]_0_/2, fewer than half of proteins are in complex). Increased concentrations on the membrane can then substantially increase complex formation. e) Similar to Fig 3D, membrane localization can act as a switch to turn on assembly from <50% to >50% (shaded areas) depending on K_a_^PP^. Here we used K_a_^PM^ = 10^4^M^-1^ and [P]_0_ = 1μM. f) Enhancement increases with smaller *σ* as is clear from Eq 3. In g) We show how mutations that would alter K_a_^PM^ (initially set here to 10^6^M^-1^) to a new value, K_a_^PM*^, would result in a change from K_a_^eff^ to K_a_^eff*^. Here we set K_a_^PP^ = 10^6^M^-1^ and [P]_0_ = 1μM. For systems with higher [M]_0_, only significant (>factor of 50) decreases in affinity due to mutation affect the enhancement. h) For Epsin and AP180, the effect of pH and mutations on lipid binding affinity have been measured experimentally[27]. We illustrate here that because these proteins target PI(4,5)P_2_ at [M]_0_ = 2.5x10^4^ μm^-2^, these up to 10-fold changes in affinity have relatively minor impact on enhancement.
(TIF)

S4 Fig Average time to reach equilibrium is shifted by membrane localization. a) ODE simulations show how localization can produce relative speed-ups and slow-downs to reach equilibrium relative to pure solution binding. The dashed line is a theoretical maximum estimated from comparing time-scales of pure 2D binding to pure 3D binding (Methods and Supplementary Text). Values of k_off_ = 1s^-1^ were used, and diffusion was only captured implicitly in binding rates, as ODEs have no spatial resolution. We started with V = 50μm^3^ and A = 65.63μm^2^, and then kept the volume constant and varied the area. K_a_^PP^ = 10^6^M^-1^, K_a_^PM^ = 10^6^M^-1^ b) For the CME binding pairs, many binding reactions are ultimately slowed by membrane localization. For human proteins, V = 1200μm^3^ and A = 767μm^2^ and for yeast proteins, V = 37.2μm^3^ and A = 75.8μm^2^ (S3 Table). c) The trend is even more evident for scaffold-mediated interactions. Same interactions as Fig 4 (S3 and S4 Datasets). d) Time-dependence of the simulations of the model in Fig 1 comparing ODES (black lines) with RD simulations using FPR [19] [20], averaged over 24 trajectories (colors). Time-scales from ODEs are similar to RD methods despite lacking explicit diffusion because our definitions of macroscopic rates implicitly account for diffusion [20]. K_a_^PP^ = 10^7^M^-1^ k_off_ = 1s^-1^, K_a_^PM^ = 2x10^6^M^-1^, [P_1_]_0_ = [P_2_]_0_ = 1μM, [M]_0_ = 17000μm^-2^. For the ODE, V = 50μm^3^ and A = 65.63μm^2^, and for the RD, we used a box size of 0.467x0.467x0.762μm, producing the same V/A ratio but in a smaller Volume. For large systems, the RD simulations will be slower to reach equilibrium due to the time needed to diffuse to the membrane. Equilibrium values of all species for this system are collected in the lower Table. e) In purely 2D simulations, we also verify that the ODE (black lines) and RD simulations (colors) give the same equilibrium, as expected. The surface area was set to of 0.467x0.467μm for the RD simulations, and the equivalent area (0.218μm^2^) for the ODEs. Time-dependence is also similar to reach that equilibrium. These results are used to define the average time-scales to equilibrate in part (f) [P_1_]_0_ = [P_2_]_0_ = 458.92μm^-2^ k_off_ = 1s^-1^ f) Pure 2D binding (green-RD or blue-ODE) is generally faster than 3D (red) despite slow-downs (factor of 100 here) in diffusion. Only for very strong (diffusion-limited) binding reactions does the impact of the diffusional search make a dominant impact on binding. V/A = 0.762, and same copy numbers in 3D and 2D, with solution concentrations of [P_1_]_0_ = [P_2_]_0_ = 1μM. Data on 2D timescales is defined from results of part (e). ODE system sizes and RD box sizes are the same as for part (d-e). Theory in black dashed (3D) and gray (2D). (g) Equilibrium values of all species for the system studied in part (d). For the stochastic FPR simulations, standard deviations from 24 trajectories are shown. Theory values are derived from Eq 3, then S1 Text Section 2A.
(TIF)

S5 Fig CME interactions assuming weaker 2D binding or fewer lipids have lower but still significant enhancements. a) If we increase σ from 1nm to 10nm, the maximal enhancement decreases by a factor of 10. b) If we reduce the lipid concentration by a factor of 10 (with σ = 1nm), enhancement again decreases relative to Fig 4. Now we are in the regime where lipids only slightly outnumber proteins. c) Complexation for pairs of (a) is still quite large, because of the overall high enhancement. Gray bars indicate complex formation without membrane present, colors are with membrane present, matching the legend in (a). d) Complexation for pairs in (b) is now much more sensitive to K_a_^PM^. Notably, when AP-2 binds cargo (light green relative to dark green bars), K_a_^PM^ is 40 times higher. The consequence of this stronger K_a_^PM^ in (c) is marginal, but with limited lipids in (d), it drives significantly larger increases in complex formation. All results in S3 Dataset.
(TIF)

S6 Fig Peripheral membrane proteins can be stabilized on the membrane via their protein-protein interactions. a) We compare FCHo1’s localization to the membrane by itself (gray bars) or with help from protein-protein interactions (colors). We consider a range of possible values for K_a_^PM^ and two different K_a_^PP^ for binding to AP-2 to show how the ability to bind other proteins will help stabilize the lipid binding FCHo1 on membranes, where we estimated [M]_0_ as 25000μm^-2^. b) The same effect is possible if the protein pairs can bind through multiple domains. Because AP-2 can use both its α and β appendages to bind epsin, it can form more complexes and stay tethered more strongly to the membrane. Results here used 10 times less PI(4,5)P_2_. Other model inputs are based on *in vivo* measurements and are collected in S3 Dataset.
(TIF)

S7 Fig Model of scaffold-mediated interactions with membrane localization. We show all the possible interactions for a system with three cytosolic proteins (P_3_, P_4_, S) and a membrane lipid (M). The two peripheral membrane proteins P_3_ and P_4_ do not directly bind one another, but both can bind to a scaffold protein S. The scaffold protein thus has two binding sites, one for P_3_ and one for P_4_. Only P_3_ and P_4_ can bind the lipid, not S. a) Binding interactions occurring purely in solution (3D) are shown in this box. b) All the orange boxed interactions involve the localization of a protein or protein complex from solution to the membrane via binding a lipid or membrane localized protein. Hence these are all 3D interactions. In panels c-f we show all the 2D interactions that can thus exploit membrane localization to enhance complex formation.
(TIF)

S8 Fig Scaffold-mediated interactions and clathrin polymerization also benefit from localization on surfaces. a) For scaffold-mediated interactions (S7 Fig), the two peripheral membrane proteins do not directly bind one another. Thus, no shift in localization will occur unless a scaffold protein bridges them. Increasing concentration of the scaffold protein increases enhancements (see S1 Text for definition of K_a_ values from simulation). Inset shows how the enhancement at a fixed V/A increases with increasing scaffold to peripheral protein. Black dashed line is maximal enhancement of K_a_^eff,SP^/K_a_^Sol,SP^ = γ. b) We simulated a system of clathrin and the adaptor AP-2 to mimic *in vitro* experiment [1] using rule-based Gillespie simulations (Methods). We extracted a V/A ratio of 9.46μm and a lipid concentration of 54,668 μm^-2^ from the study, and used clathrin and AP-2 concentrations of 0.4μM each. Stronger K_a_ (= K_d_^-1^) values for the clathrin-clathrin (CC) interaction produce more polymerization, particularly with membrane localization included (green lines). Yellow pie is percent clathrin on the membrane for simulations with AP-2. The enhancement is not limited by the AP-2:PI(4,5)P_2_ interaction, but rather because the recruitment of clathrin to the membrane requires AP-2, which is only at 0.4μM (~25 times lower than lipid at this V/A = 9.46μm). c) Clathrin binds moderately to AP-2 (22μM). However, because clathrin has three leg domains that can each bind AP-2, once on the membrane, it will quickly bind multiple AP-2s. d) Time-scales to reach equilibrium are slowed (relative to pure solution in red) due to the time needed to bind AP-2 to the membrane, and then clathrin. Simulation inputs in S4 Dataset.
(TIF)

S9 Fig Higher order assemblies studied via rule-based stochastic simulation. a) Retromer components VPS17 and VPS5 bind weakly to PI(3)P on endosomes. K_a_^PP^ values are not known for these interactions, so we estimate a range (0.1–100μM: error bars). Since the protein-lipid affinity is only known to be >100μM (26), we compare values of 100μM and weaker binding of 300μM. We compare dimerization without membrane (gray bars) and with (dark red). When assisted by an (putative) interaction between SNX3 and VPS17, more complexation occurs of the now 3-protein complexes (light red). Because SNX3 binds strongly to PI(3)P, it will drive more complexation even without VPS5 (right bars). b) VPS17 is more effectively recruited to the endosome when it also interacts with SNX3. Pink is VPS17 by itself, dark red is with dimer formation allowed, and light red is with the third protein added (SNX3 on left, VPS5 on right). Although this direct interaction between SNX3 and VPS17 is not physiological, SNX3 does bind the full 5-protein retromer complex [40]. These results illustrate how the retromer complex could be more strongly recruited to endosomes with the help of SNX3. c) We simulated BAR (SH3GL2:SH3GL2) and F-BAR (FCHo1:FCHo2) domain proteins forming both dimers and higher-order oligomers (Methods). Both pairs are given a weak oligomer binding strength of 500μM. Binding strength of proteins to the membrane is either not known or is reported at widely varying values, so we consider a range of values (0.1–100μM). Oligomerization in solution is <0.01%, but with membrane (light blue) it is especially prominent for the homodimer forming endophilin (SH3GL2). This is because SH3GL2 forms large oligomers (>20 proteins per complex) feeding back into stabilization at the membrane. In contrast, FCHo1 has much lower concentration than FCHo2, so oligomer contacts are much less likely to form large filaments. d) Dimers (no oligomer allowed) in dark blue. Simulation inputs in S4 Dataset.
(TIF)

S10 Fig Membrane localization can enhance binding for pairs of protein binding partners in diverse pathways targeting distinct organellar membranes. Yeast proteins that can also bind lipids including PI(4,5)P_2_, PI(3)P, PI(4)P, and PI(3,5)P_2_ at distinct organelles are reported in Table 1, with interactions collected in S1 Table and S2 Dataset. These proteins are involved in oxysterol binding (yellow), membrane remodeling (mauve, purple and turquoise) and vesicle assembly on endosomes (green). Most of the proteins exhibit significant enhancements, except for the endosome assembly proteins (VPS5, VPS17, SNX4, SNX41, ATG20) due to their low affinity for PI(3)P on the endosomal membrane.
(TIF)
