# Supplementary material for: Cytosolic proteins can exploit membrane localization to trigger functional assembly
Source: PLoS Comput Biol. 2018 Mar 5;14(3):e1006031. doi: 10.1371/journal.pcbi.1006031 (PMC5854442; doi:10.1371/journal.pcbi.1006031)
Supplement: S1 Text — (PDF) [file pcbi.1006031.s001.pdf]

SUPPLEMENTARY INFORMATION for

Cytosolic proteins can exploit membrane localization to trigger functional assembly

O. N. Yogurtcu and M. E. Johnson*

SUPPLEMENTARY TEXT:

1. Model Definition

1A. *Definitions of ordinary differential equations (ODEs) for the protein pair model*

We provide here the system of ODEs that describes the time evolution of all 9 species where two proteins P_1 and P_2 bind each other and the membrane lipid *M*.
$[P_1], [P_2], [M], [MP_1], [P_2M], [P_1P_2], [MP_1P_2], [P_1P_2M], [MP_1P_2M]$, where $\gamma_1=V/(2A\sigma_1)$, and σ_1 is for PP binding and σ_2 is for PM binding. We set them equal in simulations, and note that only σ_1 appears in Eq. 3 of the main text; σ_2 only appears in the definition of $[M]_{eq}^{coop}$ (see below).
These equations were solved numerically using Mathematica.

Eq. S1.1	$\frac{d[P_1]}{dt} = -k_{on}^{PP}[P_1][P_2] + k_{off}^{PP}[P_1P_2] - k_{on}^{P_1M}[P_1][M] + k_{off}^{P_1M}[MP_1] - k_{on}^{PP}[P_1][P_2M] + k_{off}^{PP}[P_1P_2M]$
Eq. S1.2	$\frac{d[P_2]}{dt} = -k_{on}^{PP}[P_1][P_2] + k_{off}^{PP}[P_1P_2] - k_{on}^{P_2M}[P_2][M] + k_{off}^{P_2M}[P_2M] - k_{on}^{PP}[P_2][MP_1] + k_{off}^{PP}[MP_1P_2]$
Eq. S1.3	$\frac{d[M]}{dt} = -k_{on}^{P_1M}[P_1][M] + k_{off}^{P_1M}[MP_1] - k_{on}^{P_2M}[P_2][M] + k_{off}^{P_2M}[P_2M] - \gamma_2 k_{on}^{P_1M}[P_1P_2M][M] - \gamma_2 k_{on}^{P_2M}[MP_1P_2][M] + k_{off}^{P_1M}[MP_1P_2M] + k_{off}^{P_2M}[MP_1P_2M] - k_{on}^{P_1M}[P_1P_2][M] - k_{on}^{P_2M}[P_1P_2][M] + k_{off}^{P_1\square}[MP_1P_2] + k_{off}^{P_2M}[P_1P_2M]$
Eq. S1.4	$\frac{d[MP_1]}{dt} = +k_{on}^{P_1M}[P_1][M] - k_{off}^{P_1M}[MP_1] + k_{off}^{PP}[MP_1P_2M] - \gamma_1 k_{on}^{PP}[MP_1][P_2M] - k_{on}^{PP}[MP_1][P_2] + k_{off}^{PP}[MP_1P_2]$
Eq. S1.5	$\frac{d[P_2M]}{dt} = +k_{on}^{P_2M}[P_2][M] - k_{off}^{P_2M}[P_2M] + k_{off}^{PP}[MP_1P_2M] - \gamma_1 k_{on}^{PP}[MP_1][P_2M] - k_{on}^{PP}[P_2M][P_1] + k_{off}^{PP}[P_1P_2M]$
Eq. S1.6	$\frac{d[MP_1P_2]}{dt} = +k_{on}^{P_1M}[P_1P_2][M] - k_{off}^{P_1M}[MP_1P_2] + k_{off}^{P_2M}[MP_1P_2M] - \gamma_2 k_{on}^{P_2M}[MP_1P_2][M] + k_{on}^{PP}[MP_1][P_2] - k_{off}^{\square P}[MP_1P_2]$
Eq. S1.7	$\frac{d[P_1P_2M]}{dt} = +k_{on}^{P_2M}[P_1P_2][M] - k_{off}^{P_2M}[P_1P_2M] + k_{off}^{P_1M}[MP_1P_2M] - \gamma_2 k_{on}^{P_1M}[P_1P_2M][M] + k_{on}^{PP}[P_1][P_2M] - k_{o\square f}^{PP}[P_1P_2M]$

Eq. S1.8	$\frac{d[P_1P_2]}{dt} = +k_{on}^{PP}[P_1][P_2] - k_{off}^{PP}[P_1P_2] + k_{off}^{P_2M}[P_1P_2M] + k_{off}^{P_1M}[MP_1P_2] - k_{on}^{P_1M}[P_1P_2][M] - k_{on}^{P_2M}[P_1P_2][M]$
Eq. S1.9	$\frac{d[MP_1P_2M]}{dt} = -k_{off}^{P_1M}[MP_1P_2M] - k_{off}^{P_2M}[MP_1P_2M] + \gamma_2 k_{on}^{P_1M}[P_1P_2M][M] + \gamma_2 k_{on}^{P_2M}[MP_1P_2][M] + \gamma_1 k_{on}^{PP}[MP_1][P_2M] - k_{off}^{PP}[MP_1P_2M]$

1B. *Protein pairs with self*

In the Methods and part 1A above, we define the system for two protein partners that are distinct (P_1 and P_2). For a homo-dimer forming protein, P_1=P_2, the system reduces to only 6 species, $[P_1], [P_1P_1], [M], [P_1M], [P_1P_1M], [MP_1P_1M]$, with the following individual equilibria.

S2.1. $P_1 + P_1 \rightleftharpoons P_1P_1$ (K_a^{PP})
S2.2. $P_1M + P_1 \rightleftharpoons P_1P_1M$ ($2K_a^{PP}$)
S2.3. $P_1 + M \rightleftharpoons P_1M$ (K_a^{PM})
S2.4. $P_1P_1 + M \rightleftharpoons P_1P_1M$ ($2K_a^{PM}$)
S2.5 $P_1P_1M + M \rightleftharpoons MP_1P_1M$ ($\frac{K_a^{PM}}{2}/(2\sigma^{PM})$)
S2.6. $P_1M + P_1M \rightleftharpoons MP_1P_1M$ ($K_a^{PP}/(2\sigma^{PP})$)

Reactions 5 and 6 are in 2D. Reactions in 2D list the 2D K_a values and thus require species be in units of A^{-1}. To solve all species in consistent units (i.e. solution concentrations: V^{-1}), the listed 2D K_a values must be multiplied by V/A. The main result, our equation for K_a^{eff} (Eq. 3), is identical for the self-pairs and the distinct pairs.

1C. *Scaffold-mediated interactions: Individual binding equilibria*

Each scaffold protein S has two binding sites, one each for peripheral membrane proteins P_3 and P_4. S does not bind to the membrane directly and P_3 and P_4 do not bind one another. Thus, the only way to exploit localization in 2D is by bridging P_3 and P_4 via S. There are 14 total species possible: $[P_3], [S], [P_4], [M], [MP_3], [MP_4], [MP_3S], [MP_4S], [P_3S], [P_4S], [P_3SP_4], [MP_3SP_4], [MP_4SP_3], [MP_3SP_4M]$
The individual equilibria are given by:

S3.1. $P_3 + S \rightleftharpoons P_3S$ ($K_a^{P_3S}$)
S3.2. $P_4 + S \rightleftharpoons P_4S$ ($K_a^{P_4S}$)
S3.3. $P_3S + P_4 \rightleftharpoons P_3SP_4$ ($K_a^{P_4S}$)
S3.4. $P_4S + P_3 \rightleftharpoons P_3SP_4$ ($K_a^{P_3S}$)
S3.5. $MP_3 + S \rightleftharpoons MP_3S$ ($K_a^{P_3S}$)
S3.6. $MP_4 + S \rightleftharpoons MP_4S$ ($K_a^{P_4S}$)
S3.7. $P_3 + M \rightleftharpoons MP_3$ ($K_a^{P_3M}$)
S3.8. $P_4 + M \rightleftharpoons MP_4$ ($K_a^{P_4M}$)

S3.9. $P_3S + M \leftrightharpoons MP_3S$ ($K_a^{P_3M}$)
S3.10. $P_4S + M \leftrightharpoons MP_4S$ ($K_a^{P_4M}$)
S3.11. $P_3SP_4 + M \leftrightharpoons MP_3SP_4$ ($K_a^{P_3M}$)
S3.12. $P_3SP_4 + M \leftrightharpoons MP_4SP_3$ ($K_a^{P_4M}$)
S3.13. $MP_3 + P_4S \leftrightharpoons MP_3SP_4$ ($K_a^{P_3S}$)
S3.14. $MP_4 + P_3S \leftrightharpoons MP_4SP_3$ ($K_a^{P_4S}$)
S3.15. $MP_3S + P_4 \leftrightharpoons MP_3SP_4$ ($K_a^{P_4S}$)
S3.16. $MP_4S + P_3 \leftrightharpoons MP_4SP_3$ ($K_a^{P_3S}$)
S3.17. $MP_3S + MP_4 \leftrightharpoons MP_3SP_4M$ ($K_a^{P_4S}/(2\sigma^{P_4S})$)
S3.18. $MP_4S + MP_3 \leftrightharpoons MP_3SP_4M$ ($K_a^{P_3S}/(2\sigma^{P_3S})$)
S3.19. $MP_3SP_4 + M \leftrightharpoons MP_3SP_4M$ ($K_a^{P_4M}/(2\sigma^{P_4M})$)
S3.20. $MP_4SP_3 + M \leftrightharpoons MP_3SP_4M$ ($K_a^{P_3M}/(2\sigma^{P_3M})$)

Here, reactions 17, 18, 19 and 20 are all in 2D and thus report the 2D K_a values.

For the case where P_3=P_4, S then has two identical and independent sites to bind P_3. As a result, from the individual equilibria listed above, every other equation is removed, leaving 10 total equilibria for 9 distinct species. Similar to section 1B above, several reactions then acquire a factor of two due to the symmetry of binding or unbinding two identical species. Specifically, Eqs. 1, 5, and 11 gain a factor of 2 multiplying K_a, and Eqs. 3, 17, and 19 gain a factor of ½ multiplying their K_a.

1D. *Scaffold-mediated interactions: $K_a^{sol, SP}$ and $K_a^{eff, SP}$.*
For the scaffold-mediated interactions, we now are tracking complex formation between three, not two proteins. To measure enhancement in complex formation from solution to solution with membrane localization, we define a complex as requiring all three proteins present. The definitions we supply are again not true equilibrium constants, either with or without membrane present ($K_a^{eff, SP}$ and $K_a^{sol, SP}$, respectively). This is distinct from the case for pairs, where the limits of K_a^{eff} produced true equilibrium constants. However, they are consistently defined with and without membrane present, such that as membrane binding reduces to zero, we have that $K_a^{eff, SP} \longrightarrow K_a^{sol, SP}$. Thus, this formulation provides a similar measure of enhancement that is measured for the pair protein system. In particular,

$$K_a^{eff,SP} = \frac{[P_3P_4]_{eq}^{sol}+[P_3P_4]_{eq}^{mem}}{([P_3]_{eq}^{sol}+[P_3]_{eq}^{mem})([P_4]_{eq}^{sol}+[P_4]_{eq}^{mem})} \quad \text{Eq. S4}$$

Now the only way to have a P_3P_4 complex is with an *S* sandwiched in between, requiring three proteins. The unbound components of the denominator thus include all the remaining species except these full three protein complexes. To be explicit, $[P_3P_4]^{sol} = [P_3SP_4]$; $[P_3P_4]^{mem} = [MP_3SP_4] + [MP_4SP_3] + [MP_3SP_4M]$; $[P_3]^{sol} = [P_3] + [P_3S]$; $[P_3]^{mem} = [MP_3] + [MP_3S]$; $[P_4]^{sol} = [P_4] + [P_4S]$; and $[P_4]^{mem} = [MP_4] + [MP_4S]$. For pure solution binding, the $K_a^{sol,SP}$ is given by:

$$K_a^{sol,SP} = \frac{[P_3P_4]_{eq}^{sol}}{([P_3]_{eq}^{sol})([P_4]_{eq}^{sol})} \qquad \text{Eq. S5}$$

using these same definitions. We emphasize that this equation is also not a true equilibrium constant, as the definitions of unbound states contain multiple species that combine in more than one way to form the bound state.

2. Theoretical Derivations

All theory derivations correspond to the pair protein model of binding (Figure 1, Eq. 5 (Methods)).

2A. *Derivation of solution and membrane species from K_a^{eff}*

From K_a^{eff}, one can directly calculate the concentrations of bound proteins, $[\text{Complexes}]_{eq} = [P_1P_2]_{eq}^{mem} + [P_1P_2]_{eq}^{sol}$, and unbound proteins at equilibrium by solving the quadratic equation $K_a^{eff}=[\text{Complexes}]_{eq} /(([P_1]_0-[\text{Complexes}]_{eq})([P_2]_0-[\text{Complexes}]_{eq}))$. The unbound protein is then simply $[P_n]_0-[\text{Complexes}]_{eq}$. However, these bound and unbound protein concentrations include both solution and lipid bound copies. To quantify the fraction of bound complexes on the membrane, we can use the exact equilibrium expressions

$$[P_1P_2]_{eq}^{mem} = K_a^{PP}(\gamma[P_1M][P_2M] + [P_1M][P_2] + [P_1][P_2M]) \qquad \text{Eq. S6}$$

and $[P_1P_2]_{eq}^{sol} = [P_1][P_2]K_a^{PP}$. Taking the ratio and using $K_a^{P_nM} = \frac{[P_nM]_{eq}}{[M]_{eq}[P_n]_{eq}}$ we then find:

$$\frac{[P_1P_2]_{eq}^{mem}}{[P_1P_2]_{eq}^{mem}+[P_1P_2]_{eq}^{sol}} = \frac{\gamma K_a^{P_1M}K_a^{P_2M}([M]_{eq})^2+[M]_{eq}\left(K_a^{P_1M}+K_a^{P_2M}\right)}{1+\gamma K_a^{P_1M}K_a^{P_2M}([M]_{eq})^2+[M]_{eq}\left(K_a^{P_1M}+K_a^{P_2M}\right)} \qquad \text{Eq. S7.}$$

which is plotted in Fig S1. The fraction of unbound proteins (P_1 or P_2) on the membrane is similarly derived giving for P_1, for example:

$$\frac{[P_1]_{eq}^{mem}}{[P_1]_{eq}^{mem}+[P_1]_{eq}^{sol}} = \frac{K_a^{P_1M}[M]_{eq}}{1+K_a^{P_1M}[M]_{eq}} \qquad \text{Eq. S8.}$$

These same equations apply for self-binding partners. Using these relations as well as Eq. 4 for $[M]_{eq}$ and the pairwise equilibrium equations (Eq. 5) from the methods, all equilibrium species can be calculated.

2B. *Derivation of critical membrane concentration, $[M]_c$ and critical value of $K_a^{PM}[M]_{eq}$*

From our equation for K_a^{eff}, we can also determine the critical lipid concentration $[M]_c$ needed to pull all proteins to the membrane and obtain close to the maximum number of complexes (Fig S3), which

occurs when $K_a^{eff} = \gamma K_a^{PP}$. $[M]_c$ must be at the very least $>[P_1]_0+[P_2]_0$, and in general will be much larger than this given finite values of K_a^{PM}. To get within ε of the maximum, we insert Eq. 3 into the following:

$$K_a^{eff} = (1-\varepsilon)\,\gamma K_a^{PP}, \qquad \text{Eq. S9}$$

to get $[M]_{eq}$. From $[M]_c = [M]_{eq} + [P_1]_0 + [P_2]_0$ we find (assuming $K_a^{P_1M} = K_a^{P_2M}$ for simplicity)

$$[M]_c = \frac{-1+2\gamma-2\gamma\epsilon+\sqrt{1-4\gamma+4\gamma^2-4\gamma^2\epsilon}}{2\gamma\epsilon\, K_a^{PM}} + [P_1]_0 + [P_2]_0 \qquad \text{Eq. S10.}$$

This gives the expected trend that with stronger affinity of proteins for the lipids, a lower concentration of lipids is needed to achieve maximum enhancement. This same approach gives us the critical value of the membrane stickiness via

$$K_a^{PM}[M]_{eq} = \frac{-1+2\gamma-2\gamma\epsilon+\sqrt{1-4\gamma+4\gamma^2-4\gamma^2\epsilon}}{2\gamma\epsilon} \qquad \text{Eq. S11}$$

showing how it varies as a function of the dimensionless geometry constant γ (Fig 3C).

2C. *Derivation of equation for* $[M]_{eq}^{coop}$

We provide here the solution to the problem where a protein with two lipid binding sites, notated as $P_{1,2}$, is in equilibrium with lipid recruiters, thus defining the $[M]_{eq}^{coop}$ used in the equation for $[M]_{eq}$ (Eq. 4). We first consider the case where both sites target the same lipid *M*. This case is analytically solvable, and the solution is almost certainly available elsewhere as well. In the second case, we consider each site targeting a distinct lipid. For this second case, we introduce an approximation to produce an analytical solution to this otherwise non-analytic problem.

For the first case, we have four pairwise equilibria, where the order of M in the complex indicates which site is bound, 1 or 2. We list all equilibrium constants here in volume units, enforcing all species are in volume units as well.

Eq. S12.1 $P_{1,2}+M \rightleftharpoons MP_{1,2}$, $(K_a^{P_1M})$

Eq. S12.2 $P_{1,2}+M \rightleftharpoons P_{1,2}M$ $(K_a^{P_2M})$

Eq. S12.3 $MP_{1,2}+M \rightleftharpoons MP_{1,2}M$ ($\gamma K_a^{P_2M}$ in Volume units)

Eq. S12.4 $P_{1,2}M+M \rightleftharpoons MP_{1,2}M$ ($\gamma K_a^{P_1M}$ in Volume units).

where $\gamma=V/(2A\sigma^{PM})$. The solution for $[M]_{eq}$ in this problem, which we define as $[M]_{eq}^{coop}$, is the root of a cubic equation. We note that $[P_{1,2}]_0$, the initial concentration of proteins with two lipid binding sites, is defined based on the full system (Fig 1) as $[P_{1,2}]_0=\min([P_1]_0, [P_2]_0)$. The cubic equation in one form is

$$[M]_0 = [M]_{eq}^{coop} + \frac{[P_{1,2}]_0[M]_{eq}^{coop}(K_a^{P_1M}+K_a^{P_2M}+2\gamma K_a^{P_1M}K_a^{P_2M}[M]_{eq}^{coop})}{1+(K_a^{P_1M}+K_a^{P_2M})[M]_{eq}^{coop}+\gamma K_a^{P_1M}K_a^{P_2M}([M]_{eq}^{coop})^2} \qquad \text{Eq. S13.}$$

For comparison, if the protein has only one lipid binding site, the equation is quadratic, $[M]_0 = [M]_{eq} + \frac{K_a^{PM}[M]_{eq}[P]_0}{1+K_a^{PM,}[M]_{eq}}$, and as expected, is independent of the geometry constant γ. The solution to the cubic equation for $[M]_{eq}^{coop}$ is the real root (of three possible) of: $a([M]_{eq}^{\square oop})^3 + b([M]_{eq}^{coop})^2 + c[M]_{eq}^{coop} + d = 0$, where $a = -\gamma K_a^{P_1M} K_a^{P_2M}$, $b = -\left(K_a^{P_1M} + K_a^{P_2M}\right) - 2\gamma[P_{1,2}]_0 K_a^{P_1M} K_a^{P_2M} + \gamma[M]_0 K_a^{P_1M} K_a^{P_2M}$, $c = -\left(K_a^{P_1M} + K_a^{P_2M}\right)[P_{1,2}]_0 + (K_a^{P_1M} + K_a^{P_2M})[M]_0 - 1$, and $d = [M]_0$.

Finally, we have the solution:

$$[M]_{eq}^{coop} = \frac{-1}{3a}(b+C+\Delta_0/C) \qquad \text{Eq. S14}$$

where $\Delta_0 = b^2 - 3ac$, $\Delta_1 = 2b^3 - 9abc + 27a^2d$, and $C = \left(\frac{1}{2}\left(\Delta_1+\sqrt{(\Delta_1)^2-4(\Delta_0)^3}\right)\right)^{1/3}$.

Eq. S14 was used for all theoretical calculations of $[M]_{eq}^{coop}$. However, because this solution Eq. S14 is based on an initial protein concentration targeting the lipids defined by $[P_{1,2}]_0$=min($[P_1]_0$, $[P_2]_0$), if the individual protein concentrations are highly unbalanced, there will be many leftover free proteins of one specie that could bind to any leftover lipids. To account for these binding interactions, one can subsequently solve for an equilibrium using initial concentrations of $[P]_{excess}$=abs($[P_1]_0$-$[P_2]_0$) and $[M]_{eq}^{coop}$, using an equation such as Eq. 7, which will provide a slightly lower value of unbound lipids, $[M]_{eq}^{coop*}$. This provides a small improvement to the final predicted value of $[M]_{eq}$ and therefore of K_a^{eff}.

While $[M]_{eq}^{coop}$ is cumbersome, we note firstly that just solving for $[M]_{eq}$ without it (Eq. 4 setting λ=0) is quite accurate in most regimes; the error increases with low recruiters and strong K_a^{PP}. Secondly, the full solution is still a matter of just plugging in known values, and requires no simulations or numerical methods. Hence it can be easily calculated via an Excel file or a Matlab script, as we provide here.

We note that if each protein targets a distinct lipid, then Eq. S13 becomes more complicated, and we have

$$[M_1]_0 = [M_1]_{eq} + \frac{[P_{1,2}]_0\left(K_a^{P_1M}[M_1]_{eq}+\gamma K_a^{P_1M}K_a^{P_2M}[M_1]_{eq}[M_2]_{eq}\right)}{\left(1+K_a^{P_2M}[M_2]_{eq}+K_a^{P_1M}[M_1]_{eq}+\gamma K_a^{P_1M}K_a^{P_2M}[M_1]_{eq}[M_2]_{eq}\right)} \qquad \text{Eq. S15}$$

and

$$[M_2]_0 = [M_2]_{eq} + \frac{[P_{1,2}]_0\left(K_a^{P_1M}[M_2]_{eq}+\gamma K_a^{P_1M}K_a^{P_2M}[M_1]_{eq}[M_2]_{eq}\right)}{\left(1+K_a^{P_2M}[M_2]_{eq}+K_a^{P_1M}[M_1]_{eq}+\gamma K_a^{P_1M}K_a^{P_2M}[M_1]_{eq}[M_2]_{eq}\right)} \qquad \text{Eq. S16}$$

Solving these simultaneously requires numerical methods. To produce a cubic root, we define an approximate relationship between M_1 and M_2. We will assume the bound states of each site, without cooperativity, is in the same proportion as the bound states of each site with cooperativity. That gives us,

$$[M_1]_{eq} = [M_1]_0 - \frac{[PM_1]_{eq}}{[PM_2]_{eq}}\left([M_2]_0 - [M_2]_{eq}\right) \qquad \text{Eq. S17}$$

where

$$[PM_1]_{eq} = \frac{1}{2}\left([M_1]_0 + [P_{1,2}]_0 + \frac{1}{K_a^{P_1M}} - \sqrt{\left([M_1]_0 + [P_{1,2}]_0 + \frac{1}{K_a^{P_1M}}\right)^2 - 4[P_{1,2}]_0[M_1]_0} \right). \quad \text{Eq. S18}$$

This transforms Eq. S16 (or Eq. S15) into a cubic equation for $[M_2]_{eq}$, that can then be solved in the same manner as the above.

2D. ***Approximation to K_a^{eff} that lacks cooperativity***

In Figure 2 of the main text, we use one other theoretical approach to predict K_a^{eff} shown with the gray lines. Instead of the derivation used to produce Eq. 3, we simply partition the proteins into solution and membrane pools based on their lipid binding strengths, K_a^{PM}, and then evaluate complexes in each subdomain. This therefore does not account for any 2D protein-lipid interactions, only 2D protein-protein interactions. This proceeds in three steps: from the initial state with all proteins unbound in solution we (1) update protein-lipid bound states assuming simple equilibrium controlled by K_a^{PM}, using an equation such as Eq. S18. We then (2) evaluate complexes formed in the solution pool as in Fig 1a (3D protein-protein) and the (3) membrane bound pool as in Fig 1c (2D protein-protein). Thus, this approximation does not capture the cooperative effect produced by bound protein-protein complexes that target the lipids (Fig 1d,e), and is therefore useful in highlighting its significance.

This method requires us to solve a sequence of three quadratic equations (such as Eq. S18) to get equilibrium concentrations first for partitioning proteins between solution and membrane, and then based on the separation of proteins between solution and membrane, solving for the protein complexes in each isolated subsystem. It has an analytical formula so the input parameters can be plugged directly into this. However, unlike Eq. 3, the resulting equations are not easy to interpret, as the results of each quadratic root must be fed into the next quadratic formula. This approach works well when the cooperative effect is less pronounced, including for weak binding proteins or when the lipid concentrations are low (Fig 2).

3. Further Simulation details

3A. ***Simulation parameters for ODEs of Figures 2 and 3.***

For the simulations of Fig 2, V=50μm^3 and V/A=0.76μm. Initial protein concentrations were $[P_1]_0$=$[P_2]_0$=2μM. For 2a, $[M]_0$=2.5x10^{4}μm^{-2}. For Fig 2b $[M]_0$=1x10^{3}μm^{-2}, K_a^{PM}=1x10^{6}M^{-1}, and k_{off}=0.1s^{-1}. For Fig 2c$_1$, $[M]_0$=1x10^{3}μm^{-2}, and for Fig 2c$_2$, protein concentrations were increased to 10μM. Fig 2d is the same as Fig 2a$_1$, except only P_1 can actually bind to lipids ($K_a^{P_2M} = 0$). In Fig 3, default values for all simulations were $[P_1]_0$=$[P_2]_0$=1μM and $[M]_0$=2.5x10^{4}μm^{-2}, K_a^{PP}=1x10^{6}M^{-1}, K_a^{PM}=1x10^{6}M^{-1}, and the other parameters were varied as noted. Because the lipid concentration is fixed in μm^{-2} units, as V/A changes, the copy numbers of lipids changes at a different rate than the copy numbers of proteins. Therefore, in Fig 3 a maximum enhancement is reached at finite V/A. For Fig 3c, we used K_a^{PM}=1x10^{4}M^{-1} and $[P_1]_0$=0.1μM, V=50μm^3 and V/A=0.76. For Fig 3d, we used $[M]_0$=1.7x10^{4}μm^{-2} and otherwise default values. For the RD simulations, we used the same V/A ratios, but the value of V was smaller (0.16μm^3) as these simulations are significantly slower to run.

3B. *RD simulation treatment of recruitment from solution to the surface*

For proteins that interact when one is bound to a lipid and the other is in solution, the interaction is still a 3D search and uses solution (3D) rates. The same on-rates are therefore applied: $k_{on}^{3D,recruit}=k_{on}^{3D}$. This is also needed to preserve detailed balance and reach an equilibrium steady-state. However, in the RD simulations, using the same intrinsic rate for this reaction produces a macroscopic rate that is ½ that of the expected k_{on}^{3D} because the proteins can only approach one another from above once one is stuck to the membrane. To preserve the equilibrium solution, the RD sims must therefore be solved with $k_a^{recruit} \neq k_a^{3D}$, but rather with $k_a^{recruit}$ defined using k_{on}^{3D} multiplied by 2 first, and then extracting k_a from Eq. 8, thus reproducing the correct $k_{on}^{3D,recruit}$ value at steady state.

4. Theoretical Background

4A. *Smoluchowski model and reactivity of binding association in 2D and 3D*

Here we provide some additional justification for the use of Eq. 13: $k_a^{2D}=k_a^{3D}/(2\sigma)$. In the well-established Smoluchowski model for binding association in all dimensions, two species diffuse and can react when they collide with one another at distance σ, which formulated mathematically results in the boundary condition(42) $D_{tot}\left.\frac{dp(r,t)}{dt}\right|_{r=\sigma}=\kappa p(\sigma,t)$. The distribution $p(r,t)$ defines the probability of finding the particles at separation r at time t. The reactivity of the surface at collision is quantified by κ in units of length/time, regardless of the dimensionality, and D_{tot} is the sum of the species' diffusion constants. We choose to preserve the reactivity κ of the reaction from 3D to 2D, as it is independent of any changes in diffusion that accompany surface restriction. Given that for a reactive sphere of radius σ in 3D, $\kappa=k_a^{3D}/(4\pi\sigma^2)$ and in 2D $\kappa=k_a^{2D}/(2\pi\sigma)$, we set both κ definitions equal and this produces Eq. 13: $k_a^{2D}=k_a^{3D}/(2\sigma)$.

4B. *Calculation of time-scales of complex formation from simulation*

To compare the time-dependence of our simulations with and without membrane recruitment, we use a mean-first passage time (MFPT) between the starting state with all proteins unbound in solution, and the final state with all proteins at equilibrium. $C(t)$ is the number of complexes present at time t, $C(0)=0$, and C_{eq} is the equilibrium complexes formed. $C(t)/C_{eq}$ is therefore the cumulative fraction of complexes formed by time t and its derivative, $\frac{1}{C_{eq}}\frac{dC(t)}{dt}$, is the probability density function describing the probability of complex formation at time t. The MFPT is then (43)

$$\langle t_c\rangle=\int_0^{\infty} t\,\frac{1}{C_{eq}}\frac{dC(t)}{dt}\,dt \qquad \text{Eq. S19.}$$

4C. *Theory estimates for time-scale bounds*

For reversible binding reactions, there is no general analytical solution for the time-dependence or characteristic time-scales even when binding is purely in solution. For irreversible association, however,

there are well-known solutions to the kinetics of A+B→C and A+A→C (see e.g. (19, 20)). We use these solutions for complexes as a function of time, $C_{\mathrm{irr}}(t)$, where $C_{\mathrm{irr}}(t)=0$ and a k_{on} is defined in either 3D or 2D. To then estimate the time-scales for *reversible a*ssociation, we use the equilibrium concentration of complexes formed, C_{eq}, defined from K_a^{PP}, and then using the irreversible formula for $C_{\mathrm{irr}}(t)$, solve for $t_{1/2}$:

$$C_{\mathrm{irr}}(t_{1/2})=C_{eq}/2 \qquad \text{Eq. S20}$$

These $t_{1/2}$ values when multiplied by two give a good estimate of the mean first passage times calculated from simulation in either 3D or 2D when the off rates are not too fast (i.e. $k_{off}=1s^{-1}$ works). While these formulas do not describe time-scales for the full system (Fig 1), they provide approximate bounds for pure solution (3D) or pure membrane bound (2D) time-scales, showing that pure 2D binding is generally significantly faster, even when k_{on} accounts for the slower diffusion on membranes (Fig S4f).

5. Biological proteins

5A. *BAR protein membrane localization*

Several of the proteins included in our interactions sets contain BAR domains, of either the F-BAR (FCHo1, FCHo2, Syp1) or N-BAR (Amphiphysin, SH3GL2/Endophilin) variety. While affinities of these domain types for membranes has been measured (50), the binding is dependent on the multi-composite nature of the membranes targeted, with each protein stabilized most likely through multiple, rather than 1:1 lipid contacts. These proteins target only negatively charged membranes containing phosphatidylserine (PS) present at some percentage via their F-BAR (16, 18, 51, 52) or N-BAR domain (11, 17, 50). The addition of $PI(4,5)P_2$ to these membranes tends to increase affinity for the membrane particularly for FCHo1 and 2, (52) and less so for other BAR domains (16, 50). Sphingolipids also play a role for the yeast N-BAR RVS proteins (11). The affinity of several BAR domains for membranes also varies with membrane curvature(17, 53), and since most of these proteins have been shown to curve and tubulate membranes *in vitro* (11, 18, 52), this creates a positive feedback influencing subsequent protein targeting to the membrane. Although this type of feedback is not present in our model, meaning our predictions are a lower bound on localization to the membrane, we note that estimating affinities of BAR domains for membranes is quite possible, although dependent on the particular membrane studied. As noted in the main text, the ratio of proteins bound to the membrane versus in solution for an *in vitro* experiment is equivalent to our membrane stickiness quantity, $K_a^{PM}[M]_{eq}$. This means it is possible to extract these values even for more complex protein-membrane interactions from *in vitro* experiments.
