# Supplementary material for: Cytosolic proteins can exploit membrane localization to trigger functional assembly
Source: PLoS Comput Biol. 2018 Mar 5;14(3):e1006031. doi: 10.1371/journal.pcbi.1006031 (PMC5854442; doi:10.1371/journal.pcbi.1006031)
Supplement: S1 Table — (PDF) [file pcbi.1006031.s003.pdf]

Table S1. Pairwise protein-protein interactions (PPIs) and affinities

	PPI (distinct pairs)[a]	Speci e	K_d^{PP} (μM)	Literature Refs[b]
1	FCHO1:AP-2 (2)	Human	Used 1, 50	PPI: PMID:25303365. Affinity not known.
2	EPN1:AP-2 (5)	Human	1 (α) & 35 (β)	PPIs and affinities: PMID:15496985&16903783.
3	PICALM:AP-2 (2)	Human	50	PPI: PMID:17713526. Affinity, homology (PICALM<-AP180), PMID:15496985.
4	PICALM:FCHO1	Human	3	PPI: PMID:22484487. Affinity, homology (PICALM<-EPS15), PMID:27237791.
5	DAB2:AP-2 (2)	Human	10	PPI: PMID:12234931. Affinity, homology (DAB2<-AP180), PMID:15496985.
6	DAB2:FCHO2	Human	3	PPI: PMID:22484487. Affinity, homology (DAB2<-EPS15), PMID:27237791.
7	DAB2:FCHO1	Human	3	PPI: PMID:22484487. Affinity, homology (DAB2<-EPS15), PMID:27237791.
8	AP180:AP-2 (2)	Human	10	PPIs and affinities: PMID:15496985.
9	ARH:AP-2 (2)	Human	2.4	PPI: PMID:12234931. Affinity, homology (ARH<-βArrestin), PMID:16516836.
10	HIP1:HIP1	Human	0.001	PPIs and affinities: PMID:18790740 Affinity estimate for stable dimers.
11	HIP1R:HIP1R	Human	0.001	PPIs and affinities: PMID:18790740. Affinity estimate for stable dimers
12	AMPH:AMPH	Human	10	PPI: PMID:22888025. Affinity, homology (AMPH<-SH3GL2), PMID:16763559.
13	SH3GL2:SH3GL2	Human	10	PPIs and affinities: PMID:16763559.
14	FCHO1:FCHO1	Human	2.5	PPI: PMID:20448150. Affinity, homology (FCHO1<-FCHO2), PMID:17540576.
15	FCHO2:FCHO2	Human	2.5	PPIs and affinities: PMID:17540576.
16	FCHO1:FCHO2	Human	2.5	PPI: PMID:20448150. Affinity, homology (FCHO1<-FCHO2), PMID:17540576.
17	SYP1:SYP1	Yeast	2.5	PPI: PMID:19713939. Affinity, homology (SYP1<-FCHO2), PMID:17540576.
18	SLA2:SLA2	Yeast	0.001	PPI: PMID:21849475. Affinity, homology (SLA2<-HIP1), PMID:18790740.
19	OSH2:SWH1 (4)	Yeast	Used 0.01, 1.0	PPI: PMID:16554755. Affinity not known.
20	OSH2:OSH2 (4)	Yeast	Used 0.01, 1.0	PPI: PMID:22940862. Affinity not known.
21	SWH1:SWH1 (4)	Yeast	Used 0.01, 1.0	PPI: PMID:16554755,18467557. Affinity not known.
22	KES1:KES1 (2)	Yeast	Used 0.01, 1.0	PPI: PMID:22940862. Affinity not known.
23	VPS17:SNX4	Yeast	Used 0.01, 1.0	PPI: PMID:15263065. Affinity not known.
24	SNX4:SNX41	Yeast	Used 0.01, 1.0	PPI: PMID:22875988, 16554755, 16429126, 12554655, 20826334, 15263065, 11283351,19591838. Affinity not known.
25	VPS5:VPS17	Yeast	Used 0.01, 1.0	PPI: PMID:16554755, 22940862, 11598206, 9285823, 9700157, 18467557, 12181349, 10688190, 15263065, 17696874. Affinity not known.
26	ATG20:SNX4	Yeast	Used 0.01, 1.0	PPI: PMID:16429126, 12048214, 12554655, 18467557, 22615397, 10688190, 15263065, 18719252, 19591838. Affinity not known.
27	BOI2:CLA4 (3)	Yeast	Used 0.01, 1.0	PPI: PMID:11489916, 8666672. Affinity not known.
28	CLA4:SKM1 (2)	Yeast	Used 0.01, 1.0	PPI: PMID:14660704. Affinity not known.
29	BOI2:BEM1	Yeast	1.0	PPI: PMID:8666672. Affinity, homology (SH3/PRD interaction PMID14668868, PMID15834155, PMID19590096).
30	BOI1:BEM1	Yeast	1.0	PPI: PMID:8666672. Affinity, homology (SH3/PRD interaction PMID14668868, PMID15834155, PMID19590096).
31	BEM1:CLA4	Yeast	Used 0.01, 1.0	PPI: PMID:19841731, 22277653, 21489982, 21118957, 11113154. Affinity not known.
32	VAM7:BEM1	Yeast	Used 0.01, 1.0	PPI: PMID:16854988. Affinity not known.
33	SLA2:CLA4	Yeast	Used 0.01, 1.0	PPI: PMID:11489916. Affinity not known.

a) Repeated enhancement calculations because one or both protein bound multiple lipid types, or proteins bound each other through multiple domain pairs.
b) See Datasets S2, S3 for further details, rates, and all results.
