# Supplementary material for: Cytosolic proteins can exploit membrane localization to trigger functional assembly
Source: PLoS Comput Biol. 2018 Mar 5;14(3):e1006031. doi: 10.1371/journal.pcbi.1006031 (PMC5854442; doi:10.1371/journal.pcbi.1006031)
Supplement: S2 Table — (PDF) [file pcbi.1006031.s004.pdf]

Table S2. Scaffold-mediated PPIs and higher-order assemblies, with affinities

	PPI (distinct sets)[a]	Species	K_d^{PS} (μM)	Literature Refs	K_d^{SP} (μM)	Literature Refs[b]
1	AP-2A:ITSN1:DAB2	Human	11	PPI and affinity: PMID: 15496985.	0.07	PPI: PMID:12234931. Affinity, homology (DAB:ITSNpartners on PRD): PMID:15834155.
2	AP-2A:ITSN1:FCHO1	Human	11	PPI and affinity: PMID: 15496985.	3	PPI: PMID:20448150. Affinity, homology (FCHO1:EPS15): PMID:27237791.
3	FCHO1:ITSN1:DAB2	Human	3	PPI: PMID: 20448150. Affinity, homology (EPS15:FCHO1): PMID:27237791.	0.07	PPI: PMID:14596919. Affinity, homology (DAB:ITSN partners on PRD): PMID:15834155.
4	AP-2:EPS15:AP-2 (2)	Human	0.021&18	PPI and affinity: PMID:15496985&16903783.	0.021&18	PPI and affinity: PMID:15496985&16903783.
5	AP-2:EPS15:EPN1 (2)	Human	0.021&18	PPI and affinity: PMID:15496985&16903783.	90	PPI and affinity: PMID: 18200045.
6	AP-2:EPS15:FCHO1 (2)	Human	0.021&18	PPI and affinity: PMID:15496985&16903783.	3	PPI and affinity: PMID: 27237791.
7	ENT1:EDE1:SYP1	Yeast	90	PPI: PMID:12529323, 18448668. Affinity, homology (Human EPN:EPS15): PMID: 18200045.	3	PPI: PMID:19713939, 19776351. Affinity, homology (Human FCHO:EPS15): PMID: 27237791.
8	ENT2:EDE1:SYP1	Yeast	90	PPI: PMID:12529323. Affinity, homology (Human EPN:EPS15): PMID: 18200045.	3	PPI: PMID:19713939, 19776351. Affinity, homology (FCHO:EPS15): PMID: 27237791.
9	SLA2:SLA1:SYP1	Yeast	Used 0.1, 10, 100	PPI: PMID:12734398. Affinity not known.	0.1	PPI: PMID:19841731. Affinity, homology (SH3-PRD interaction of dynamin with endocytic partners): PMID: 15834155.
10	SNX3:VPS5:VPS17 (Retromer Proxy)	Yeast	Used 0.01, 1.0	PPI: PMID: 17892535. Affinity not known.	Used 0.01, 1.0	PPI: PMID:16554755, 22940862, 11598206, 9285823, 9700157, 18467557, 12181349, 10688190, 15263065, Affinity not known.
	**Oligomers:**		**K_d Dimer**		**K_d Oligo**	
11	SH3GL2:SH3GL2:SH3GL2 Etc.	Human	10	PPIs and affinities: PMID:16763559.	500	PPI: PMID: 20448150. Affinity: PMID: 17540576 (Estimated weaker 130μM, minimal solution oligomers)
12	FCHo1:FCHo2:FCHo1.. Etc.	Human	2.5	PPI: PMID:20448150. Affinity, homology (FCHO1<-FCHO2), PMID:17540576.	500	PPI: PMID: 20448150. Affinity: PMID: 17540576 (Estimated weaker 130μM, minimal solution oligomers)
	**Polymerization:**		**K_d AP-2:CLTC**		**K_d CLC:CLC**	
13	AP-2:CLATH:CLATH.. Etc.	Human	22	PPI: PMID:10944104. Affinity, homology (AP-2<-AMPH): PMID:14981508.	Used 1, 10, 100, 1000	PPI: PMID: 26496610. Affinity not known.

a) Repeated enhancement calculations because proteins bound each other through multiple domain pairs. b) See Dataset S4 for further details, rates, and all results.
