# Supplementary material for: Cytosolic proteins can exploit membrane localization to trigger functional assembly
Source: PLoS Comput Biol. 2018 Mar 5;14(3):e1006031. doi: 10.1371/journal.pcbi.1006031 (PMC5854442; doi:10.1371/journal.pcbi.1006031)
Supplement: S3 Table — (PDF) [file pcbi.1006031.s005.pdf]

Table S3. Volume and membrane surface area estimates for different cells and organelles.

Compartment Type	Reaction Volume (μm^3)	Total Volume (μm^3)	Surface Area (μm^2)	V/A (μm)	Technique/Evidence
**Convex Plasma Membr.**					
3T3 Mouse Fibroblast	1200	2000	768	1.6	V:Coulter counter, SA: estimate[a]
Human Chondrocyte	990	1650	730	1.4	CM, stereology[b]
Guinea pig Pancreatic Exocrine	636	1060	581	1.1	TEM, stereology[c]
Rabbit Chondrocyte	1163	1939	1155	1.0	CM, stereology[d]
Human Erythrocyte*	54	90	136	0.4	Estimate[e]
Euglena	5	7.8	7.2	0.7	Estimate: phylogeny+published data[f]
S. Cerevisiae	37	62	75.4	0.5	V:Coulter counter, SA: estimate[g]
Human Neutrophil	120	200	300	0.4	TEM, stereology[h]
Human Lymphocyte	69	115	260	0.3	TEM, stereology[h]
Lamprey Motor Neuron	3578	5964	23430	0.2	CM, stereology[i]
E. Coli	1	1.0	6.0	0.1	Estimate[j]
**Convex Organelle**					
Rat Hepatocyte Nucleus IM	204	204	135	1.5	Hepatocyte nucleus has 6% of total cell volume[k,l]
3T3 MVB IV	11	22	38	0.3	Assuming 1% of Cytosol volume[m], ½ MVB OM SA
S. Cerevisiae MVB IM	0.6	1.2	3.8	0.2	Assuming 1% of Cytosol volume[m], ½ MVB OM SA
S. Cerevisiae VAC IM	1.0	2.1	9.0	0.1	6% of cytosol volume[n], assume ½ VAC OM SA
3T3 MIT IM	400	400	9222	0.04	Hepatocyte MIT VOL 20% of total cell volume, MIT IM SA 1200% of PM SA[k,l]
S. Cerevisiae MIT IM	12.4	12.4	905	0.01	Hepatocyte MIT VOL 20% of total cell volume, MIT IM SA 1200% of PM SA[k,l]
**Concave Organelle**					
3T3 MVB OM	1200	-	77	15.6	Assuming MVB SA 10% of PM SA
3T3 RE	1200	-	77	15.6	Assuming total RE SA 10% of PM SA
3T3 EE	1200	-	77	15.6	Assuming total EE SA 10% of PM SA
3T3 LYS	1200	-	154	7.8	Hepatocyte LYS SA 20% of PM SA[k,l]
S. Cerevisiae EE	37	-	7.5	4.9	Assuming EE SA 10% of PM SA
S. Cerevisiae MVB OM	37	-	7.5	4.9	Assuming EE SA 10% of PM SA
S. Cerevisiae VAC OM	37	-	18	2.1	CM, image analysis
3T3 MIT OM	1200	-	1921	0.6	Hepatocyte MIT OM SA 2.5 times of PM SA [k,l]
3T3 Golgi	1200	-	2690	0.4	Hepatocyte Golgi SA 3.5 times of PM SA [k,l]
S. Cerevisiae MIT OM	37	-	189	0.2	Hepatocyte MIT OM SA 2.5 times of PM SA [k,l]
S. Cerevisiae Golgi	37	-	264	0.1	Hepatocyte Golgi SA 3.5 times of PM SA [k,l]
3T3 ER	1200	-	13832	0.1	Hepatocyte ER SA 18 times of PM SA[k]
S. Cerevisiae ER	37	-	1358	0.03	Hepatocyte ER SA 18 times of PM SA[k]

(*)Cytoplasmic volume assumed to be the same as total cell volume. Abbreviations: EE-Early endosomes, RE-Recycling endosomes, IV-Intraluminal vesicles of endosomes, VAC-Vacuoles, G-Golgi, PM-Plasma membrane, NUC-Nucleus, ER-Endoplasmic reticulum, LE-Late endosomes, LYS-Lysosomes, MVB-Multivesicular bodies, MIT-Mitochondria, OM-Outer membrane, IM-Inner membrane, CM-Confocal microscopy, TEM-transmission electron microscopy. **(a)** PMID1366595 & PMID1614815 **(b)** PMID15970445 **(c)** PMID4363955 **(d)** DOI: 10.1111/j.1365-2818.1994.tb03447.x **(e)** PMID3565597 **(f)** PMID19443453 **(g)** PMID12089449 **(h)** PMID6775712 **(i)** DOI:10.1006/ncmn.1993.1016 **(j)** PMID14681416,ISBN: 9781134111589 **(k)** ISBN: 9780815344322 **(l)** PMID833203 **(m)** PMID862008 **(n)** PMID27151661.
