# Supplementary material for: Cytosolic proteins can exploit membrane localization to trigger functional assembly
Source: PLoS Comput Biol. 2018 Mar 5;14(3):e1006031. doi: 10.1371/journal.pcbi.1006031 (PMC5854442; doi:10.1371/journal.pcbi.1006031)
Supplement: S4 Table — (PDF) [file pcbi.1006031.s006.pdf]

**Table S4. Phosphoinositide ($PtdInsP_n$) and phosphatidylserine concentrations across various organelles in a mammalian and a yeast cell.**

| | % Lipid | #Lipid 3T3 | SA 3T3 ($\mu m^2$) | Lipid Conc. 3T3 ($\mu m^{-2}$) | Notes | #Lipid *S.C.* | SA *S.C.* ($\mu m^2$) | Lipid Conc. *S.C.* ($\mu m^{-2}$) | Notes |
|---|---|---|---|---|---|---|---|---|---|
| **PtdIns(3)P** | | **Total lipids:** 1.69 $10^6$ | | | | **Total lipids:** 2.79 $10^6$ | | | |
| MVB IV (3T3) | 20%[a] | 3.4 $10^5$ | 38 | 2.94 $10^4$ | Total lipids calculated from ratio to $PI(4,5)P_2$[j] | - | - | - | Set total lipids of PI(3)P to same as total $PI(4,5)P_2$[k],[l] |
| VAC OM (B.Y.) | 41%[a] | - | - | - | | 1.14 $10^6$ | 18 | 6.3 $10^4$ | |
| VAC IM (B.Y.) | 41%[a] | - | - | - | | 1.14 $10^6$ | 9 | 1.27 $10^5$ | |
| Cyt Vesicles (B.Y.) | 9%[a] | - | - | - | | 2.51 $10^5$ | 8 | 3.3 $10^4$ | |
| **PtdIns(4)P** | | **Total lipids:** 3.73 $10^7$ | | | | **Total lipids:** 2.79 $10^6$ | | | |
| G | 45%[b] | 1.68 $10^7$ | 2,687 | 6.25 $10^3$ | PM total lipids from (SAxPM Lipid Conc.) Total lipids from fraction on PM. | 1.25 $10^6$ | 265 | 4.7 $10^3$ | Set total lipids of PI(4)P to same as total $PI(4,5)P_2$[k],[l] |
| PM | 35%[b] | 1.31 $10^7$ | 768 | 1.7 $10^4$ [i] | | 9.75 $10^5$ | 76 | 1.29 $10^4$ | |
| LYS | 15%[b] | 5.6 $10^6$ | 154 | 3.64 $10^4$ | | 4.18 $10^5$ | 18 | 2.32 $10^4$ | |
| EE | 10%[b] | 3.73 $10^6$ | 77 | 4.86 $10^4$ | | 2.8 $10^5$ | 8 | 3.68 $10^4$ | |
| **PtdIns(5)P** | | **Total lipids:** 1.19 $10^6$ | | | | **Total lipids:** Undetected | | | |
| PM | 27%[c] | 3.2 $10^5$ | 768 | 420 [i] | PM total lipids from (SAxPM Lipid Conc.) Total lipids from fraction on PM. | - | - | - | |
| **$PtdIns(3,4)P_2$** | | **Total lipids:** 8.06 $10^4$ | | | | **Total lipids:** Undetected | | | |
| PM | 40%[d] | 3.2 $10^4$ | 768 | 42 [i] | PM total lipids from (SAxPM Lipid Conc.) Total lipids from fraction on PM. | - | - | - | |
| **$PtdIns(3,5)P_2$** | | **Total lipids:** 2.64 $10^5$ | | | | **Total lipids:** 2. $10^5$ | | | |
| LYS/VAC | 85%[e] | 4.8 $10^5$ | 153.7 | 3.1 $10^3$ | Total lipids calculated from ratio to $PI(4,5)P_2$[j] | 1.69 $10^5$ | 18 | 9.4 $10^3$ | Set total lipids of $PI(3,5)P_2$ 14x lower than total $PI(4,5)P_2$[k],[l],[m] |
| **$PtdIns(4,5)P_2$** | | **Total lipids:** 2.82 $10^7$ | | | | **Total lipids:** 2.79 $10^6$ | | | |
| PM | 68%[f] | 1.92 $10^7$ | 768 | 2.5 $10^4$ [h] | PM total lipids from (SAxPM Lipid Conc.) Total lipids from fraction on PM. | 1.89 $10^6$ | 75.4 | 2.5 $10^4$ | Used same PM lipid conc. as 3T3[h], then see Notes for $PI(4,5)P_2$ 3T3. |
| MVB OM | 4%[f] | 1.13 $10^6$ | 77 | 1.47 $10^4$ | | 1.11 $10^5$ | 7.5 | 1.47 $10^4$ | |
| MVB IV | 4%[f] | 1.13 $10^6$ | 38 | 2.94 $10^4$ | | 1.11 $10^5$ | 3.8 | 2.94 $10^4$ | |
| **$PtdIns(3,4,5)P_3$** | | **Total lipids:** 3.07 $10^4$ | | | | **Total lipids:** Undetected | | | |
| PM | 70%[g] | 2.15 $10^4$ | 768 | 28 [i] | PM total lipids from (SAxPM Lipid Conc.) Total lipids from fraction on PM. | - | - | - | |
| RE | 25%[g] | 7.68 $10^3$ | 77 | 100 | | - | - | - | |
| **PhosphatidylSerine** | | | | | | | | | |
| PM | NA | 9.6 $10^7$ | 768 | 1.25 $10^5$ | Set PM total lipids to 5x that of PM total $PI(4,5)P_2$[n] | 9.47 $10^6$ | 76 | 1.25 $10^5$ | Set PM total lipids to 5x that of PM total $PI(4,5)P_2$[n] |

Abbrev: 3T3/NIH fibroblast cells, (*S.C.*) *Saccharomyces Cerevisiae.* SA Surface Area. Compartment abbreviations same as Table S1. **(a)** PMID10970851 **(b)** PMID24711504 **(c)** PMID20370717 **(d)** PMID14604433 **(e)** PMID24324172 **(f)** PMID11964166 **(g)** PMID19864464, PMID25345859. **(h)** PMID22024883 **(i)** Ref.(13) **(j)** PMID22621786 **(k)** PMID11854411 **(l)** PMID17392273. **(m)** PMID11889142 **(n)** ~9% of lipids on PM are PS: PMID24007978, 1-1.5% are $PI(4,5)P_2$.
