# Supplementary figures and images for: Cytosolic proteins can exploit membrane localization to trigger functional assembly
Source: PLoS Comput Biol. 2018 Mar 5;14(3):e1006031. doi: 10.1371/journal.pcbi.1006031 (PMC5854442; doi:10.1371/journal.pcbi.1006031)

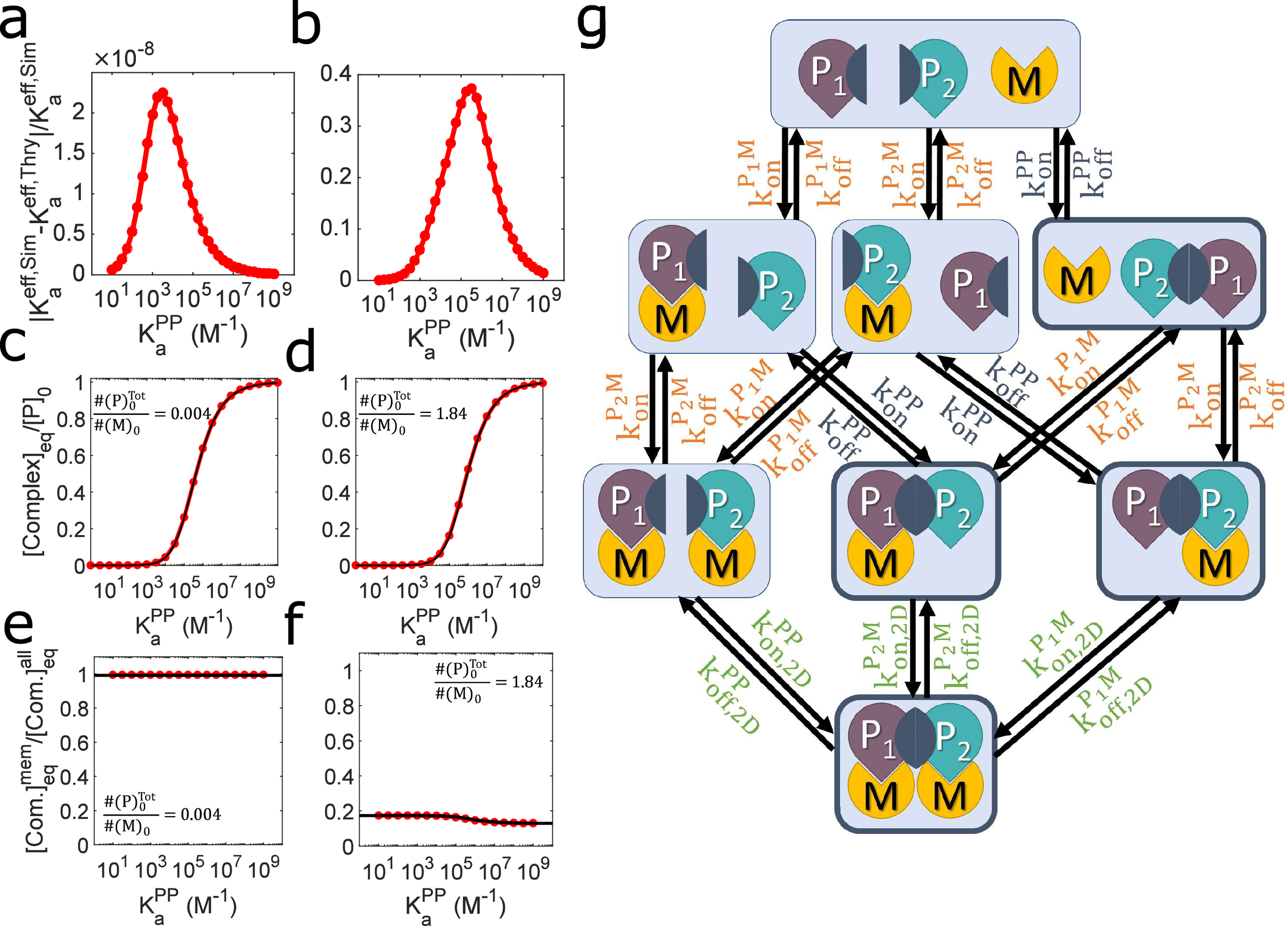

Supplement: S1 Fig — a,b) The relative error of the theoretically predicted Kaeff values compared with the exact numerical result from simulation, with data corresponding to the results of Fig 2A1 and 2C1, respectively. As expected, the error (~10−8) is negligible in (a) under the conditions of excess lipids simulated in Fig 2A1, as [M]eq is nearly exactly predicted by our approximate theory. In (b), the error increases now that lipids are outnumbered. Error is highest for moderate KaPP values, because here the predicted value of [M]eq is farthest from either of the limiting (and exact) predictions of [M]eq0 or [M]eqCoop. The error reduces to values of 10−3 and 10−2 near these limits. c,d) From Eq 3 Kaeff, we can directly calculate the concentration of bound protein-protein complexes, as Kaeff = [Complex]eq/(([P1]0-[Complex]eq)([P2]0-[Complex]eq)). Simulation (red) vs theory (black). c) V/A = 0.76, KaPM = 104M-1, [P1]0 = [P2]0 = 0.1 μM, [M]0 = 2.5104 μm−2. (d) Same as (c) except [P1]0 = [P2]0 = 2 μM and [M]0 = 103 μm−2 e,f) The fraction of these complexes that are specifically on the membrane (Eq. S7). g) Network of reactions between all states. States with black outline all contain protein-protein complexes. Reactions in 2D are in green text. Protein-protein binding is otherwise in navy text, and protein-lipid binding in orange text. (TIF) [file pcbi.1006031.s011.tif]

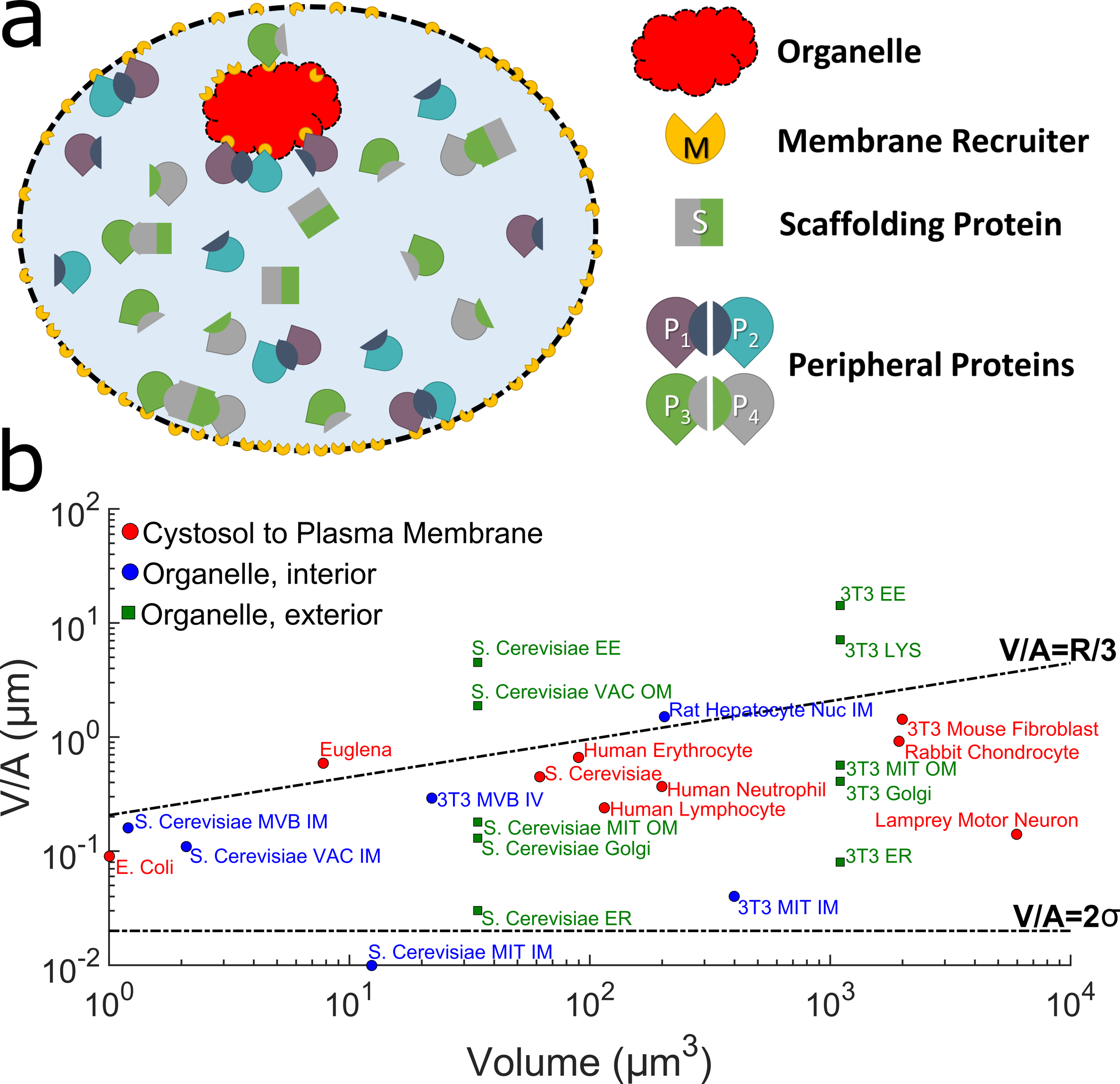

Supplement: S2 Fig — a) Proteins in the cytosol can localize to membranes by binding specific lipids (yellow). Peripheral membrane proteins that do not bind directly can be bridged by a scaffold protein (green/gray) b) We collected the solution volumes (V) and membrane surface areas (A) for both plasma and organellar membranes in a variety of cell types (S3 Table). Only when the V/A ratio drops below 2σ, where here σ is set to 10nm, does the membrane reduce binding relative to solution (bottom black line). The only case found here is for proteins inside the yeast Mitochondria, which has a small volume but a large surface area due to the highly invaginated structure of the membrane. The V/A ratio for a sphere (V/A = R/3) is shown for reference in the diagonal black line. (TIF) [file pcbi.1006031.s012.tif]

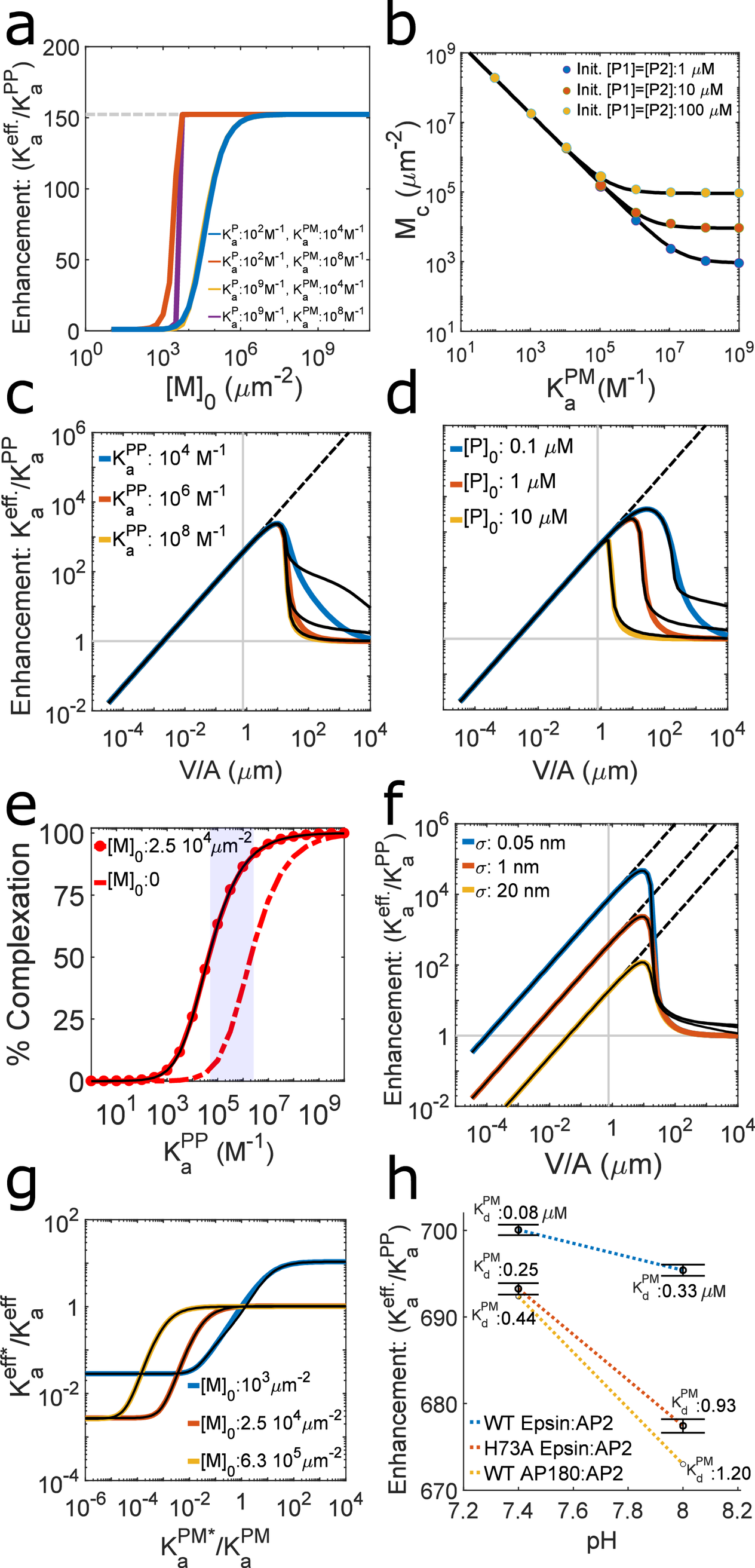

Supplement: S3 Fig — a) Once the enhancement due to membrane localization is near to the maximum value, the addition of more lipids changes the binding equilibrium imperceptibly. Even a relatively low concentration of lipids is needed to trigger the maximum binding interactions, particularly with strong KaPM. b) This critical lipid concentration, [M]c, beyond which no further changes are observed in binding is derived in S1 Text (simulation results are points, theory is lines). We define maximum binding as within ε of Kaeff = γKaPP, with results here shown for ε = 0.01. c) Protein interactions between proteins with weak solution binding (low KaPP) or d) low protein concentrations benefit more widely from recruitment. This is because these systems will form minimal complexes in solution (with (KaPP)-1>[P]0/2, fewer than half of proteins are in complex). Increased concentrations on the membrane can then substantially increase complex formation. e) Similar to Fig 3D, membrane localization can act as a switch to turn on assembly from <50% to >50% (shaded areas) depending on KaPP. Here we used KaPM = 104M-1 and [P]0 = 1μM. f) Enhancement increases with smaller σ as is clear from Eq 3. In g) We show how mutations that would alter KaPM (initially set here to 106M-1) to a new value, KaPM*, would result in a change from Kaeff to Kaeff*. Here we set KaPP = 106M-1 and [P]0 = 1μM. For systems with higher [M]0, only significant (>factor of 50) decreases in affinity due to mutation affect the enhancement. h) For Epsin and AP180, the effect of pH and mutations on lipid binding affinity have been measured experimentally[27]. We illustrate here that because these proteins target PI(4,5)P2 at [M]0 = 2.5x104 μm-2, these up to 10-fold changes in affinity have relatively minor impact on enhancement. (TIF) [file pcbi.1006031.s013.tif]

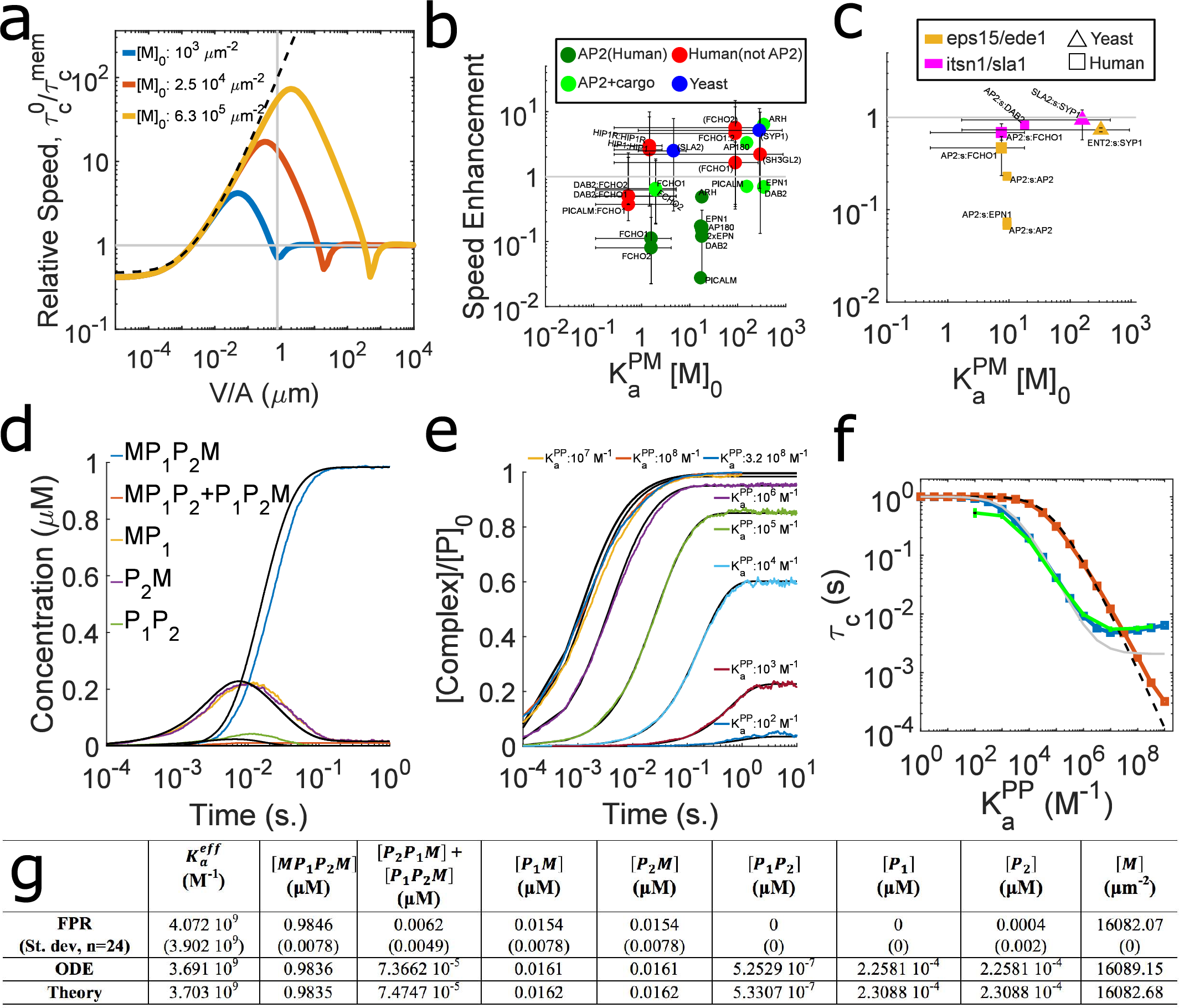

Supplement: S4 Fig — a) ODE simulations show how localization can produce relative speed-ups and slow-downs to reach equilibrium relative to pure solution binding. The dashed line is a theoretical maximum estimated from comparing time-scales of pure 2D binding to pure 3D binding (Methods and Supplementary Text). Values of koff = 1s-1 were used, and diffusion was only captured implicitly in binding rates, as ODEs have no spatial resolution. We started with V = 50μm3 and A = 65.63μm2, and then kept the volume constant and varied the area. KaPP = 106M-1, KaPM = 106M-1 b) For the CME binding pairs, many binding reactions are ultimately slowed by membrane localization. For human proteins, V = 1200μm3 and A = 767μm2 and for yeast proteins, V = 37.2μm3 and A = 75.8μm2 (S3 Table). c) The trend is even more evident for scaffold-mediated interactions. Same interactions as Fig 4 (S3 and S4 Datasets). d) Time-dependence of the simulations of the model in Fig 1 comparing ODES (black lines) with RD simulations using FPR [19] [20], averaged over 24 trajectories (colors). Time-scales from ODEs are similar to RD methods despite lacking explicit diffusion because our definitions of macroscopic rates implicitly account for diffusion [20]. KaPP = 107M-1 koff = 1s-1, KaPM = 2x10^6^M-1, [P1]0 = [P2]0 = 1μM, [M]0 = 17000μm-2. For the ODE, V = 50μm3 and A = 65.63μm2, and for the RD, we used a box size of 0.467x0.467x0.762μm, producing the same V/A ratio but in a smaller Volume. For large systems, the RD simulations will be slower to reach equilibrium due to the time needed to diffuse to the membrane. Equilibrium values of all species for this system are collected in the lower Table. e) In purely 2D simulations, we also verify that the ODE (black lines) and RD simulations (colors) give the same equilibrium, as expected. The surface area was set to of 0.467x0.467μm for the RD simulations, and the equivalent area (0.218μm2) for the ODEs. Time-dependence is also similar to reach that equilibrium. These results are u [file pcbi.1006031.s014.tif]

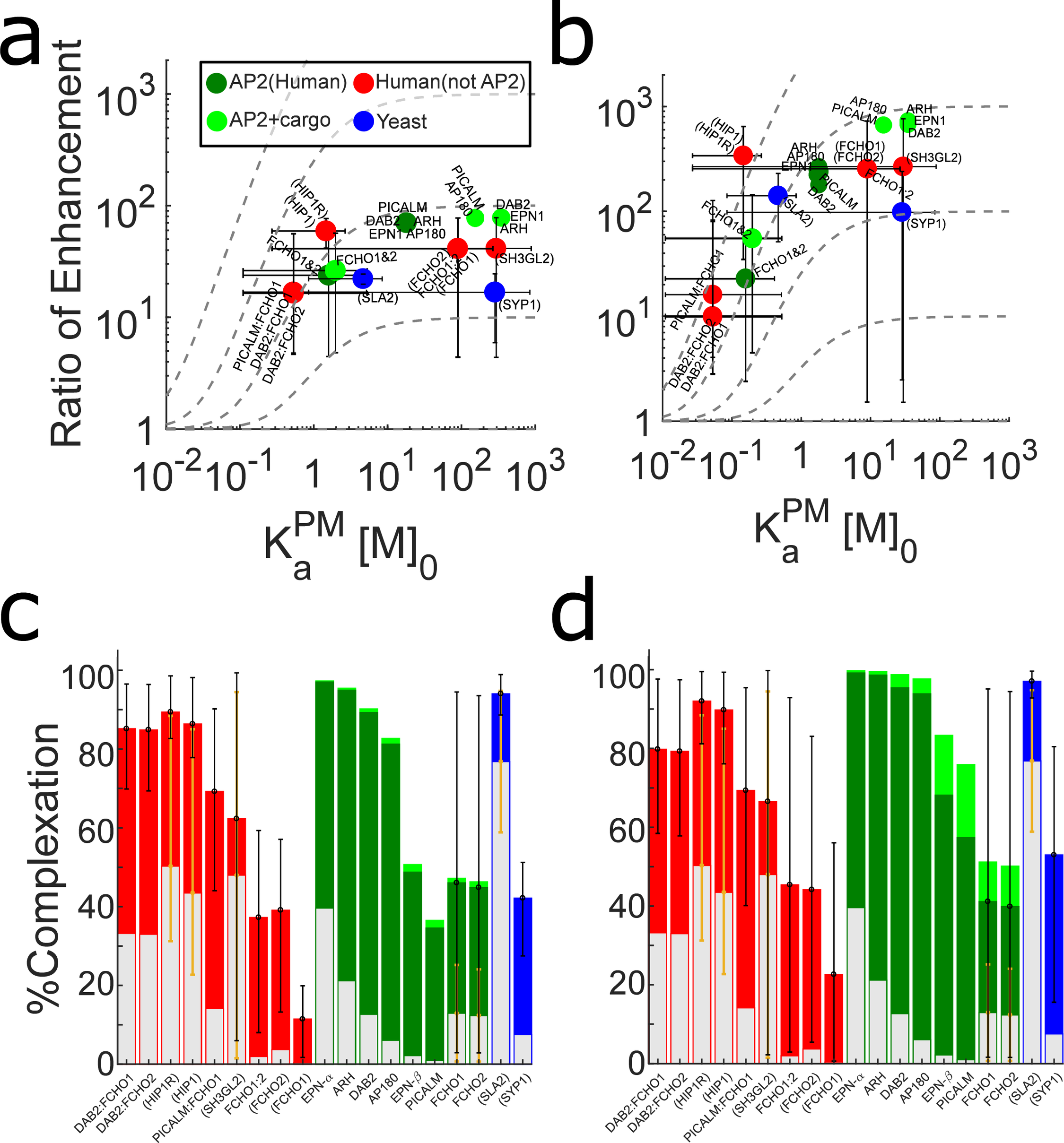

Supplement: S5 Fig — a) If we increase σ from 1nm to 10nm, the maximal enhancement decreases by a factor of 10. b) If we reduce the lipid concentration by a factor of 10 (with σ = 1nm), enhancement again decreases relative to Fig 4. Now we are in the regime where lipids only slightly outnumber proteins. c) Complexation for pairs of (a) is still quite large, because of the overall high enhancement. Gray bars indicate complex formation without membrane present, colors are with membrane present, matching the legend in (a). d) Complexation for pairs in (b) is now much more sensitive to KaPM. Notably, when AP-2 binds cargo (light green relative to dark green bars), KaPM is 40 times higher. The consequence of this stronger KaPM in (c) is marginal, but with limited lipids in (d), it drives significantly larger increases in complex formation. All results in S3 Dataset. (TIF) [file pcbi.1006031.s015.tif]

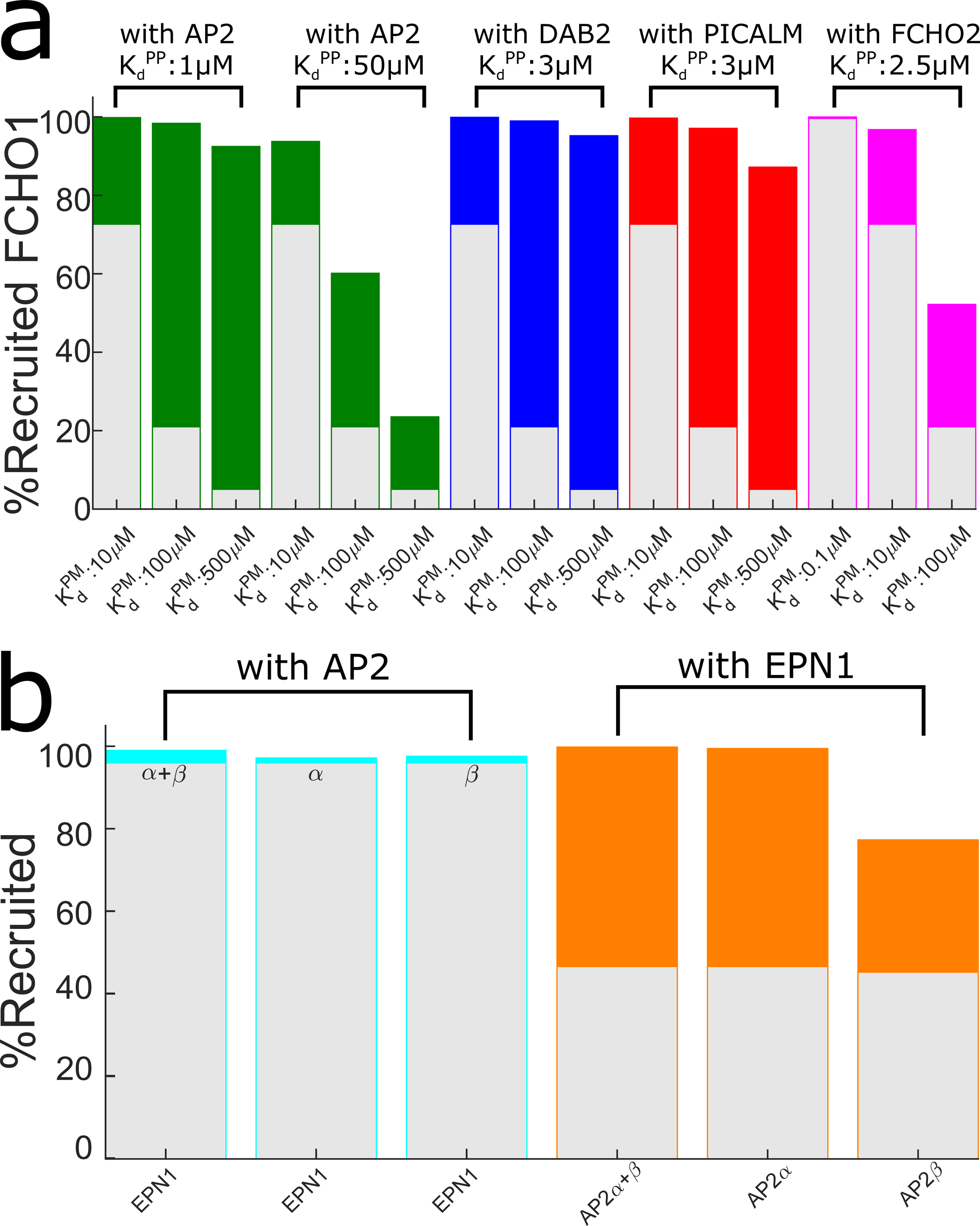

Supplement: S6 Fig — a) We compare FCHo1’s localization to the membrane by itself (gray bars) or with help from protein-protein interactions (colors). We consider a range of possible values for KaPM and two different KaPP for binding to AP-2 to show how the ability to bind other proteins will help stabilize the lipid binding FCHo1 on membranes, where we estimated [M]0 as 25000μm-2. b) The same effect is possible if the protein pairs can bind through multiple domains. Because AP-2 can use both its α and β appendages to bind epsin, it can form more complexes and stay tethered more strongly to the membrane. Results here used 10 times less PI(4,5)P2. Other model inputs are based on in vivo measurements and are collected in S3 Dataset. (TIF) [file pcbi.1006031.s016.tif]

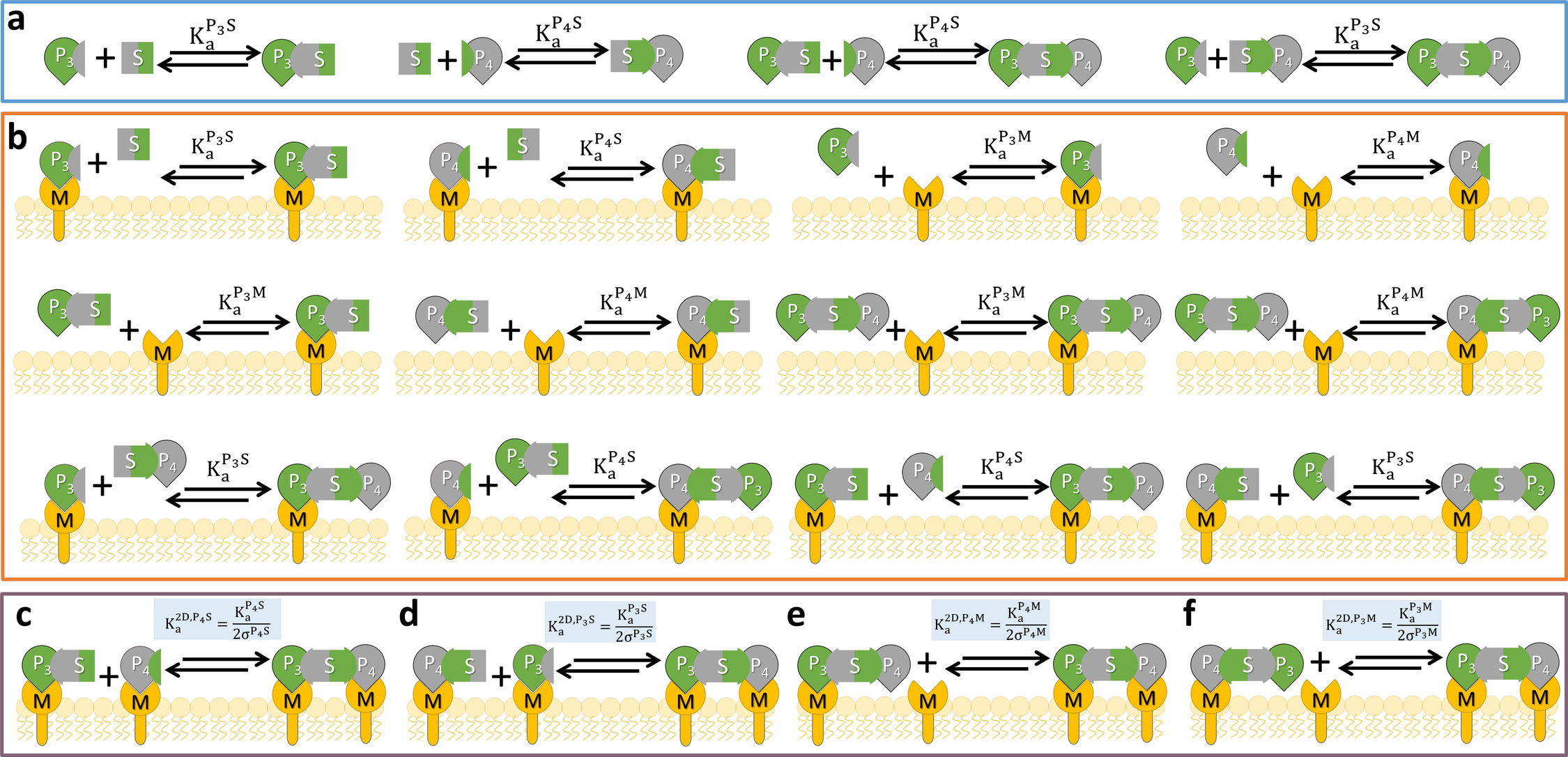

Supplement: S7 Fig — We show all the possible interactions for a system with three cytosolic proteins (P3, P4, S) and a membrane lipid (M). The two peripheral membrane proteins P3 and P4 do not directly bind one another, but both can bind to a scaffold protein S. The scaffold protein thus has two binding sites, one for P3 and one for P4. Only P3 and P4 can bind the lipid, not S. a) Binding interactions occurring purely in solution (3D) are shown in this box. b) All the orange boxed interactions involve the localization of a protein or protein complex from solution to the membrane via binding a lipid or membrane localized protein. Hence these are all 3D interactions. In panels c-f we show all the 2D interactions that can thus exploit membrane localization to enhance complex formation. (TIF) [file pcbi.1006031.s017.tif]

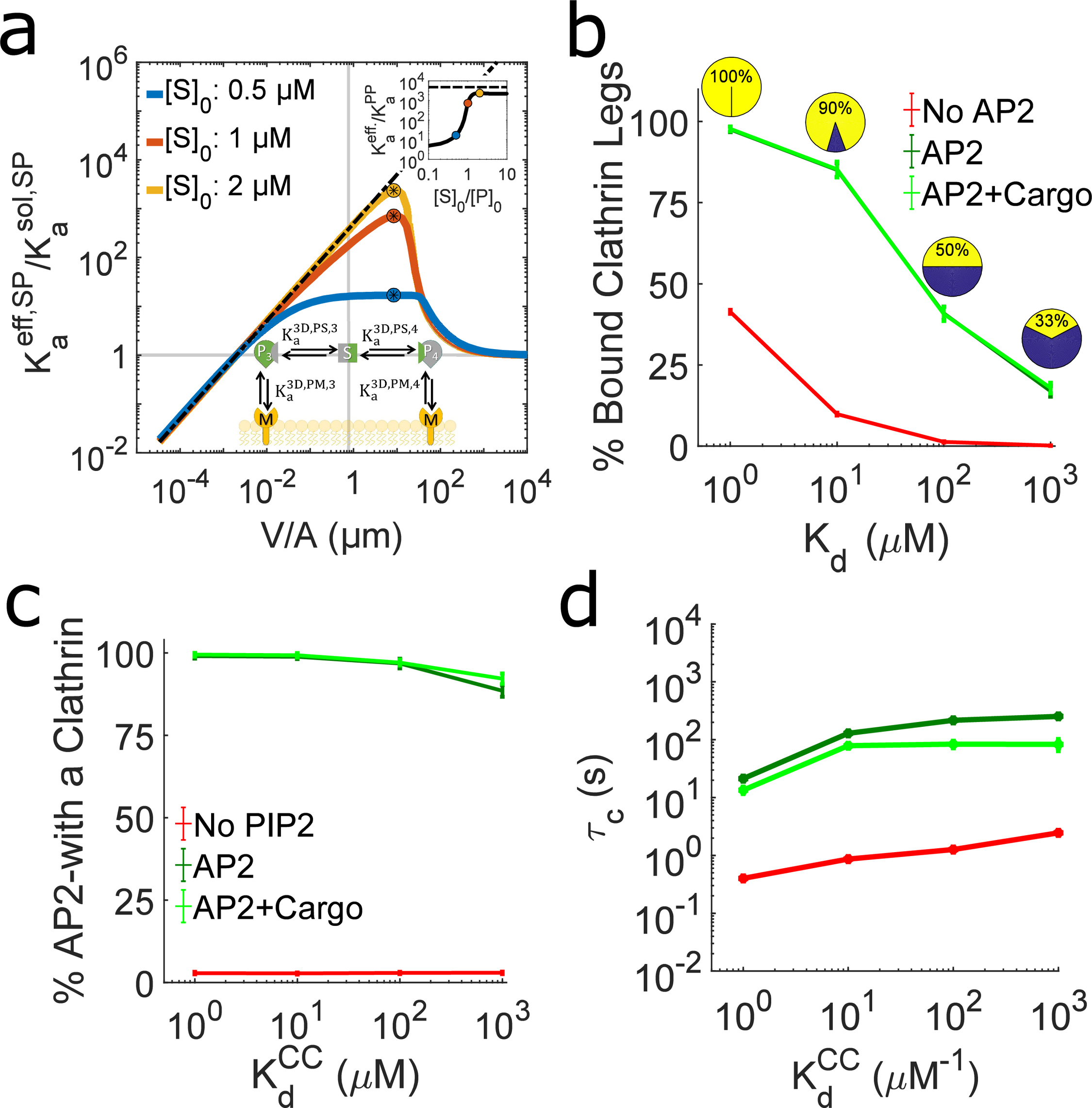

Supplement: S8 Fig — a) For scaffold-mediated interactions (S7 Fig), the two peripheral membrane proteins do not directly bind one another. Thus, no shift in localization will occur unless a scaffold protein bridges them. Increasing concentration of the scaffold protein increases enhancements (see S1 Text for definition of Ka values from simulation). Inset shows how the enhancement at a fixed V/A increases with increasing scaffold to peripheral protein. Black dashed line is maximal enhancement of Kaeff,SP/KaSol,SP = γ. b) We simulated a system of clathrin and the adaptor AP-2 to mimic in vitro experiment [1] using rule-based Gillespie simulations (Methods). We extracted a V/A ratio of 9.46μm and a lipid concentration of 54,668 μm-2 from the study, and used clathrin and AP-2 concentrations of 0.4μM each. Stronger Ka (= Kd-1) values for the clathrin-clathrin (CC) interaction produce more polymerization, particularly with membrane localization included (green lines). Yellow pie is percent clathrin on the membrane for simulations with AP-2. The enhancement is not limited by the AP-2:PI(4,5)P2 interaction, but rather because the recruitment of clathrin to the membrane requires AP-2, which is only at 0.4μM (~25 times lower than lipid at this V/A = 9.46μm). c) Clathrin binds moderately to AP-2 (22μM). However, because clathrin has three leg domains that can each bind AP-2, once on the membrane, it will quickly bind multiple AP-2s. d) Time-scales to reach equilibrium are slowed (relative to pure solution in red) due to the time needed to bind AP-2 to the membrane, and then clathrin. Simulation inputs in S4 Dataset. (TIF) [file pcbi.1006031.s018.tif]

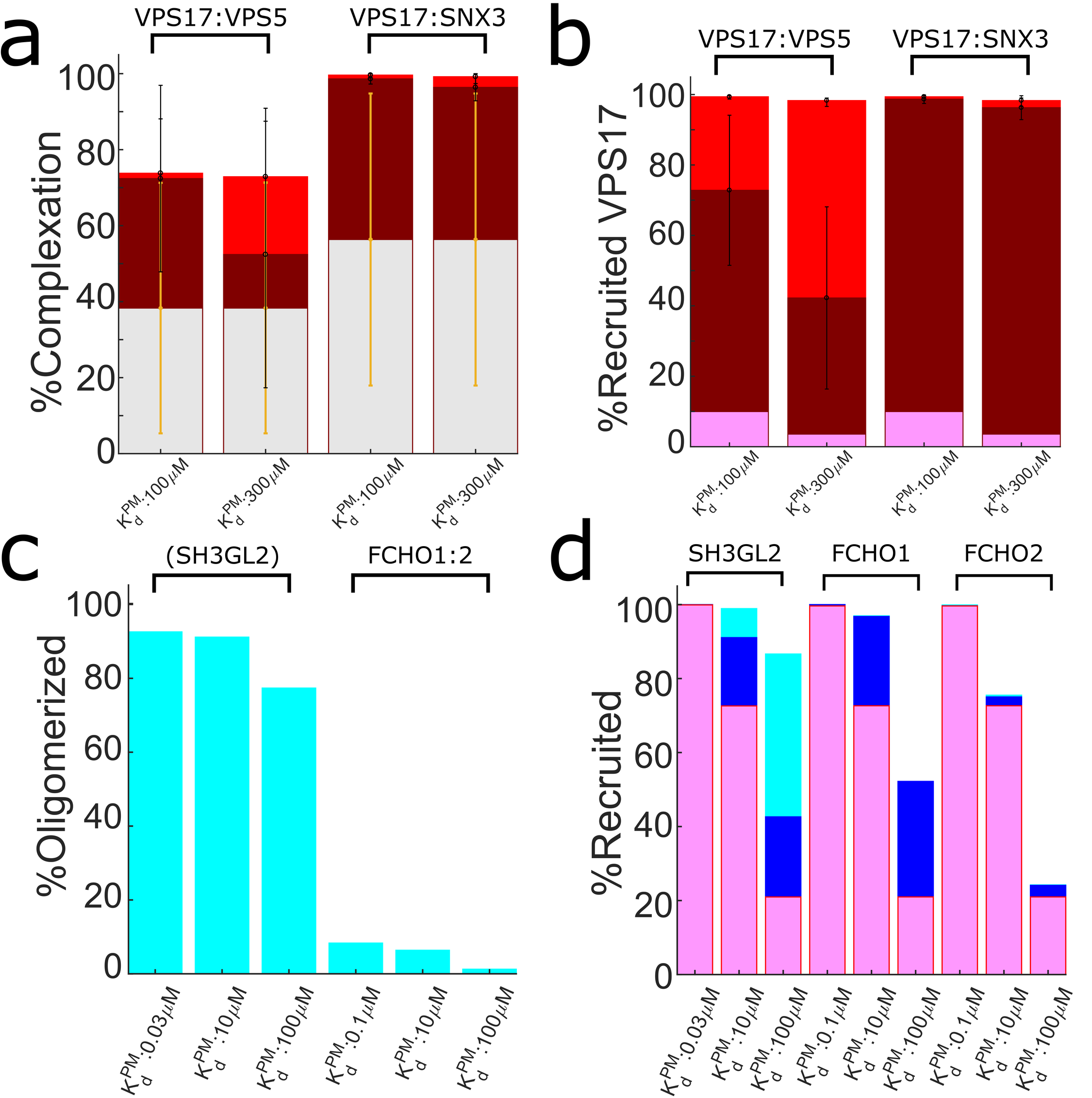

Supplement: S9 Fig — a) Retromer components VPS17 and VPS5 bind weakly to PI(3)P on endosomes. KaPP values are not known for these interactions, so we estimate a range (0.1–100μM: error bars). Since the protein-lipid affinity is only known to be >100μM (26), we compare values of 100μM and weaker binding of 300μM. We compare dimerization without membrane (gray bars) and with (dark red). When assisted by an (putative) interaction between SNX3 and VPS17, more complexation occurs of the now 3-protein complexes (light red). Because SNX3 binds strongly to PI(3)P, it will drive more complexation even without VPS5 (right bars). b) VPS17 is more effectively recruited to the endosome when it also interacts with SNX3. Pink is VPS17 by itself, dark red is with dimer formation allowed, and light red is with the third protein added (SNX3 on left, VPS5 on right). Although this direct interaction between SNX3 and VPS17 is not physiological, SNX3 does bind the full 5-protein retromer complex [40]. These results illustrate how the retromer complex could be more strongly recruited to endosomes with the help of SNX3. c) We simulated BAR (SH3GL2:SH3GL2) and F-BAR (FCHo1:FCHo2) domain proteins forming both dimers and higher-order oligomers (Methods). Both pairs are given a weak oligomer binding strength of 500μM. Binding strength of proteins to the membrane is either not known or is reported at widely varying values, so we consider a range of values (0.1–100μM). Oligomerization in solution is <0.01%, but with membrane (light blue) it is especially prominent for the homodimer forming endophilin (SH3GL2). This is because SH3GL2 forms large oligomers (>20 proteins per complex) feeding back into stabilization at the membrane. In contrast, FCHo1 has much lower concentration than FCHo2, so oligomer contacts are much less likely to form large filaments. d) Dimers (no oligomer allowed) in dark blue. Simulation inputs in S4 Dataset. (TIF) [file pcbi.1006031.s019.tif]

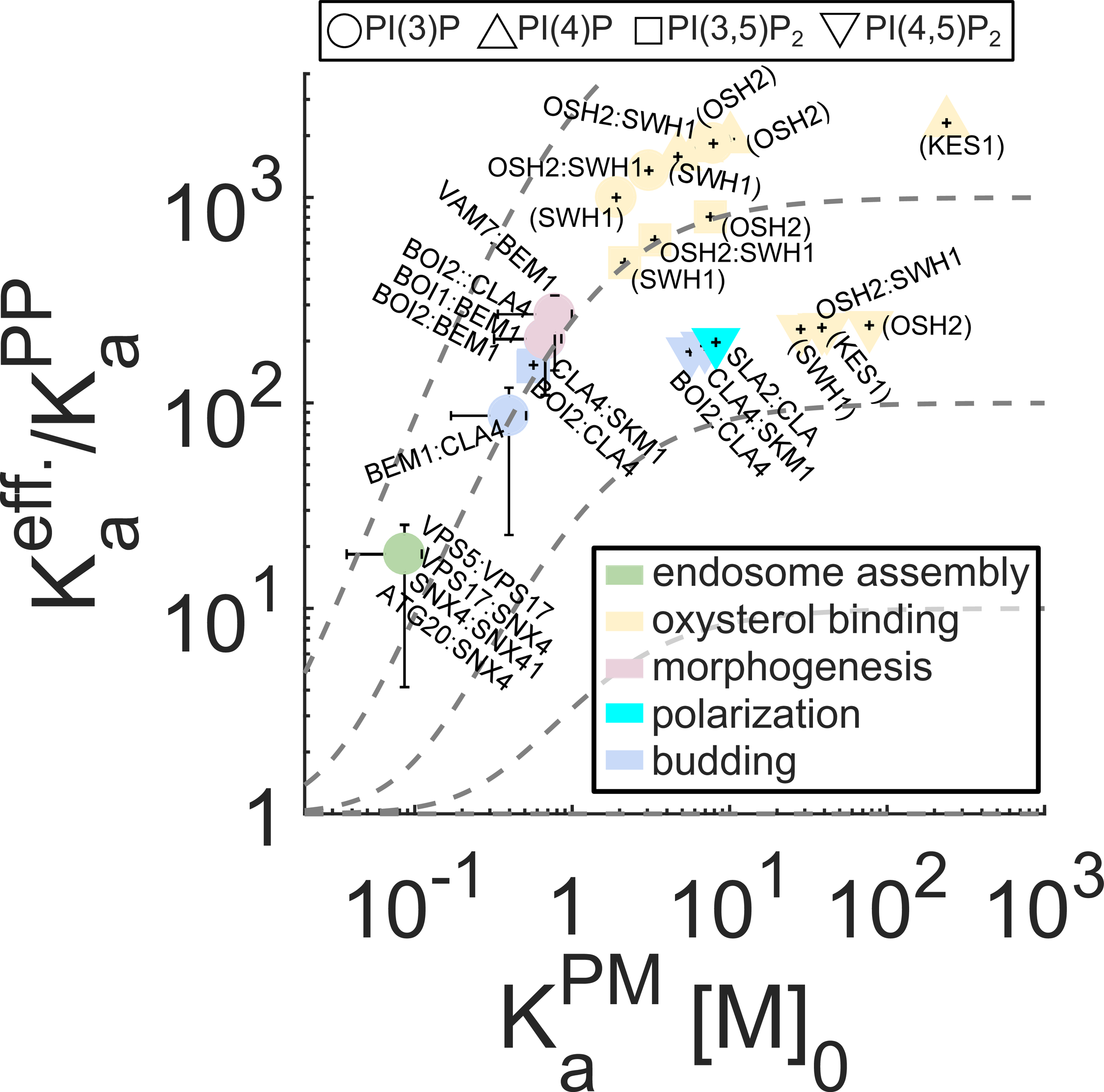

Supplement: S10 Fig — Yeast proteins that can also bind lipids including PI(4,5)P2, PI(3)P, PI(4)P, and PI(3,5)P2 at distinct organelles are reported in Table 1, with interactions collected in S1 Table and S2 Dataset. These proteins are involved in oxysterol binding (yellow), membrane remodeling (mauve, purple and turquoise) and vesicle assembly on endosomes (green). Most of the proteins exhibit significant enhancements, except for the endosome assembly proteins (VPS5, VPS17, SNX4, SNX41, ATG20) due to their low affinity for PI(3)P on the endosomal membrane. (TIF) [file pcbi.1006031.s020.tif]
